# Phosphoglycerate mutase 5 exacerbates alcoholic cardiomyopathy in male mice by inducing prohibitin‐2 dephosphorylation and impairing mitochondrial quality control

**DOI:** 10.1002/ctm2.1806

**Published:** 2024-08-14

**Authors:** Jun Tao, Junxiong Qiu, Junmeng Zheng, Ruibing Li, Xing Chang, Qingyong He

**Affiliations:** ^1^ Department of Cardiovascular Surgery Sun Yat‐sen Memorial Hospital Sun Yat‐sen University Guangzhou China; ^2^ Department of Clinical Laboratory Medicine The First Medical Centre, Medical School of Chinese People's Liberation Army Beijing China; ^3^ Xianning Medical College Hubei University of Science & Technology, Xianning, China Xianning China; ^4^ Department of Cardiology, Guang'anmen Hospital China Academy of Chinese Medical Sciences Beijing China

**Keywords:** alcoholic cardiomyopathy, MQC, Pgam5, Phb2

## Abstract

**Background:**

The induction of mitochondrial quality control (MQC) mechanisms is essential for the re‐establishment of mitochondrial homeostasis and cellular bioenergetics during periods of stress. Although MQC activation has cardioprotective effects in various cardiovascular diseases, its precise role and regulatory mechanisms in alcoholic cardiomyopathy (ACM) remain incompletely understood.

**Methods:**

We explored whether two mitochondria‐related proteins, phosphoglycerate mutase 5 (Pgam5) and prohibitin 2 (Phb2), influence MQC in male mice during ACM.

**Results:**

Myocardial Pgam5 expression was upregulated in a male mouse model of ACM. Notably, following ACM induction, heart dysfunction was markedly reversed in male cardiomyocyte‐specific *Pgam5* knockout (*Pgam5^cKO^*) mice. Meanwhile, in alcohol‐treated male mouse‐derived neonatal cardiomyocytes, *Pgam5* depletion preserved cell survival and restored mitochondrial dynamics, mitophagy, mitochondrial biogenesis and the mitochondrial unfolded protein response (mtUPR). We further found that in alcohol‐treated cardiomyocyte, Pgam5 binds Phb2 and induces its dephosphorylation at Ser91. Alternative transduction of phospho‐mimetic (Phb2^S91D^) and phospho‐defective (Phb2^S9A^) Phb2 mutants attenuated and enhanced, respectively, alcohol‐related mitochondrial dysfunction in cardiomyocytes. Moreover, transgenic male mice expressing *Phb2^S91D^* were resistant to alcohol‐induced heart dysfunction.

**Conclusions:**

We conclude that ACM‐induced Pgam5 upregulation results in Pgam5‐dependent Phb2^S91^ dephosphorylation, leading to MQC destabilisation and mitochondrial dysfunction in heart. Therefore, modulating the Pgam5/Phb2 interaction could potentially offer a novel therapeutic strategy for ACM in male mice.

**Highlights:**

- *Pgam5* knockout attenuates alcohol‐induced cardiac histopathology and heart dysfunction in male mice.
- *Pgam5* KO reduces alcohol‐induced myocardial inflammation, lipid peroxidation and metabolic dysfunction in male mice.
- Pgam5 depletion protects mitochondrial function in alcohol‐exposed male mouse cardiomyocytes.
- Pgam5 depletion normalises MQC in ACM.
- EtOH impairs MQC through inducing Phb2 dephosphorylation at Ser91.
- Pgam5 interacts with Phb2 and induces Phb2 dephosphorylation.
- Transgenic mice expressing a Ser91 phospho‐mimetic Phb2 mutant are resistant to ACM.

## Graphical abstract

- *Pgam5* knockout attenuates alcohol‐induced cardiac histopathology and heart dysfunction in male mice.
- Pgam5 interacts with Phb2 and induces Phb2 dephosphorylation.
- Disruption of Pgam5/Phb2 binding reduces EtOH‐induced mitochondrial quality control impairment in cardiomyocytes.
- Mice genetically modified to express a phosphor‐mimetic Phb2 mutant at serine 91 exhibit resistance to the development of alcoholic cardiomyopathy.

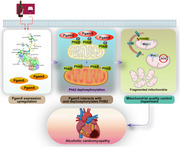

## 1 INTRODUCTION

Alcoholic cardiomyopathy (ACM) is a common type of heart ailment marked by impaired cardiac function and structural changes due to long‐term alcohol consumption. As a treatment enigma, since no targeted drugs are currently available, abstinence seems to be the only therapeutic option for patients with ACM. Hence, a better understanding of the pathological mechanisms underlying ACM is urgently needed to foster the development of novel pharmacological interventions. Multiple molecular processes, such as ethanol (EtOH) toxicity, oxidative stress, inflammation, metabolic remodelling and DNA damage, have been proposed to contribute to the pathogenesis of ACM.^1^, ^2^, ^3^, ^4^ Among these, mitochondrial malfunction has emerged as a pivotal event during its onset and progression.^5^

Exposure to stress triggers inherent mitochondrial quality control (MQC) processes, encompassing fission/fusion dynamics, mitophagy, biogenesis and the mitochondrial unfolded protein response (mtUPR). Fusion facilitates the exchange of mitochondrial components, while mtUPR restores protein homeostasis.^6^ Fission and subsequent mitophagy selectively remove damaged mitochondria,^7^ followed by organelle replenishment through biogenesis. Prior research suggests a potential involvement of mitochondrial fission and mitophagy in the pathogenesis of alcohol‐induced hepatic injury.^8^, ^9^ In cardiac tissue subjected to chronic alcohol exposure, a substantial increase in mitochondrial Drp1 levels was observed, concomitant with a decrease in Mfn2 and Opa1 expression.^10^ This dysregulation of mitochondrial dynamics proteins could potentially facilitate the release of cytochrome c, thereby triggering the initiation of apoptotic cell death pathways.^10^ Conversely, augmenting mitophagic activity via supplementation with alpha‐lipoic acid, a potent antioxidant, has been shown to confer cardioprotection against the deleterious effects of chronic alcohol consumption by restoring the PINK1/Parkin pathway.^11^ Additionally, it was reported that PGC1α‐mediated mitochondrial biogenesis is also inhibited and contributes to oxidative stress and tissue injury during alcohol‐induced cardiotoxicity.^12^ Transmission electron microscopy analysis of myocardial biopsies from individuals diagnosed with ACM demonstrated substantial ultrastructural abnormalities.^13^ Within representative areas, the contractile apparatus of the myocardium was either entirely absent or exhibited signs of degeneration, accompanied by a marked increase in the number of fragmented mitochondria.^13^ The mitochondria were swollen, and their cristae were reduced in number or completely disappeared ('mitochondrial empty shells').^13^ While these findings point to abnormal MQC functioning in ACM, the upstream regulators involved have not been fully investigated.

Phosphoglycerate mutase 5 (Pgam5) regulates necrotic cell death triggered by Fas ligand or TNFα signalling.^14^ Investigations utilising a murine model of cardiac post‐ischemic injury have elucidated the deleterious contribution of Pgam5 to myocardial damage, manifested by cardiomyocyte pyroptosis and cardiac inflammation.^15^ Moreover, Pgam5 inhibition prevented calcium‐mediated mitochondrial permeability transition pore (mPTP) opening.^16^ Furthermore, Pgam5 inhibition was shown to retard heart failure progression via a mechanism involving Keap1/Nrf2‐mediated ferroptosis.^17^ While these findings highlight Pgam5's detrimental effects in various cardiac pathologies, its contribution to ACM development remains unexplored.

Prohibitin‐2 (Phb2) is an inner mitochondrial membrane (IMM)‐localised protein that plays an indispensable role in sustaining mitochondrial stability through forming an alternating heterodimeric ring‐like complex with Phb1. Recent evidence elucidated the involvement of Phb2 in the regulation of mitophagy,^18^ mitochondrial division,^19^, ^20^ biogenesis,^21^ proteolysis^22^ and cell death^23^ in various disease models. Consistent with these reports, our recent investigations have revealed Phb2 as a previously unrecognised upstream modulator of MQC, influencing mitochondrial fission/fusion, bioenergetics and mitophagy.^24^ Although a series of studies has uncovered multiple and protective roles played by Phb2 in type‐3 cardiorenal syndrome,^25^ cardiac remodeling,^26^ heart ageing^27^ and myocardial fibrosis,^28^ it remains unknown whether Phb2 also confers cardioprotection in ACM through normalisation of MQC.

In the present investigation, we sought to elucidate the potential contributions of Pgam5 and Phb2 in ACM by evaluating the effects of cardiomyocyte‐specific *Pgam5* deletion and transgenic expression of a phospho‐mimetic mutant of Phb2 on cardiac structure and function in a mouse model of ACM. It is well documented that women are at a higher risk of developing alcohol‐related diseases compared to men.^29^, ^30^ The underlying mechanisms for this increased susceptibility in women are multifaceted and likely involve various factors, including differences in alcohol metabolism, hormonal influences, genetic predisposition and immune response variations.^31^, ^32^, ^33^, ^34^ To specifically investigate the influence of Pgam5/Phb2 on myocardial dysfunction caused by alcohol exposure, we utilised male mice, thereby excluding known sex‐based phenotypic differences in response to alcohol. Moreover, we performed in vitro assays using ethanol‐exposed neonatal cardiomyocytes derived from male mice to figure out the impact of Pgam5/Phb2 in MQC.

## 2 MATERIALS AND METHODS

### 2.1 Animals

All animal experimentation adhered to the guidelines sanctioned by the Sun Yat‐sen Memorial Hospital. Given documented sex‐based variances in answer to alcohol intake,^35^ male mice were exclusively utilised for this investigation. *Pgam5^f/f^* mice, generated as previously detailed,^36^ were bred with *α‐MHC^Cre+^* mice, to yield cardiomyocyte‐specific *Pgam5* knockout (*Pgam5^cKO^*) mice. *Phb2^S91D^* knockin mice on a C57BL/6 background were engineered by Cyagen Biosciences (Santa Clara, CA, USA). For the in vivo modelling of ACM, 8‐week‐old mice were initially adapted to a Lieber‐DeCarli liquid control diet (F1259SP; Bio‐Serv) for 5 days. Subsequently, animals were pair‐fed for 8 weeks with either the same control diet or an isocaloric diet containing 5% (v/v) ethanol (F1258SP; Bio‐Serv). Upon completion of the dietary intervention, mice were gavaged with either maltose dextrin (control; 9 g/kg body weight) or ethanol (5 g/kg body weight) and humanely sacrificed 8 h later. Tissues were promptly harvested and processed for further analysis, either by snap‐freezing in liquid nitrogen or fixation in paraformaldehyde. An echocardiogram was used to assess heart function.

### 2.2 Histological, immunohistochemical and immunofluorescent analyses

Cardiac tissue samples were preserved in 10% neutral buffered formalin, followed by dehydration and embedding in paraffin wax. Haematoxylin and eosin (H&E) staining was used for the evaluation of cardiac architecture. Picro‐Sirius red staining was utilised to visualise myocardial fibrosis. Tissue sections were examined and imaged via light microscopy (Olympus, Tokyo, Japan). Sirius red‐positive areas were quantified using Image‐Pro Plus software, with results presented as a proportion of the total analysed area. TUNEL and Nissl staining were performed to assess histological changes in hepatic, renal and cerebral tissues, respectively.

To visualise cellular structures via immunofluorescence, samples were fixed in 4% paraformaldehyde for 15 min at 4°C. Following fixation, samples were incubated overnight at 4°C with primary antibodies, and then washed extensively with Tris‐buffered saline (TBS). Subsequently, samples were incubated with a secondary antibody (Goat anti‐mouse IgG Alexa Fluor 555, 1:500; #4409; Cell Signaling Technology) and counterstained with DAPI to label nuclei. The antibodies utilised in this study are listed in Supplementary Table S1.

### 2.3 Biochemistry

Serum triglyceride (TG) levels, as well as the activity of alanine aminotransferase (ALT) and aspartate aminotransferase (AST), were assessed using an ADVIA 2400 Chemistry System analyser (Siemens). TG content in cardiac tissues and cells were quantified with a commercially available kit (290‐63701; Wako Chem.).

### 2.4 Cell isolation and treatments

Neonatal cardiomyocytes were extracted from the hearts of male WT mice or from male *Pgam5^f/f^*, *Pgam5^cKO^* and *Phb2^S91D^* mice as detailed previously.^37^ These isolated cardiomyocytes were exposed to either 100 mM ethanol or PBS for 48 h prior to analysis. Furthermore, HL‐1 cells underwent adenoviral transfection in the presence of either 100 mM ethanol or PBS for a 48‐h period.

### 2.5 Cell viability and mPTP assays

Neonatal cardiomyocyte viability was assessed via a Cell Counting Kit‐8 (CCK‐8; Dojindo). The rate of mPTP opening was estimated as previously detailed.^38^ Briefly, control and ethanol‐exposed cardiomyocytes were treated with tetramethylrhodamine ethyl ester (TMRE; 10 nM) for 30 min in the absence of light. Subsequently, TMRE fluorescence intensity was monitored every 15 s using a Nikon confocal microscope. A time‐dependent curve of fluorescence intensity changes was generated, and the time to 50% reduction in TMRE fluorescence (relative to the control group) was calculated to estimate the mPTP opening rate.^39^

### 2.6 Reactive oxygen species measurements

Mitochondrial superoxide levels were assessed by incubating neonatal cardiomyocytes with 5 mM MitoSOX Red, a fluorogenic probe specific for this reactive oxygen species (ROS) at 37°C for 25 min.^40^ The fluorescence signal, corresponding to the level of mROS, was then quantified using confocal microscopy. ImageJ software was employed for subsequent data analysis and processing.

### 2.7 Enzyme‐linked immunosorbent assay

The quantification of various cardiomyocyte and serum biomarkers was achieved through the utilisation of enzyme‐linked immunosorbent assay (ELISA) kits. These kits, supplied by MyBioSource, Inc. (San Diego, CA, USA), were employed to measure the levels of superoxide dismutase (SOD; #MBS034842), glutathione (GSH; #MBS267424), malondialdehyde (MDA; #MBS741034), glutathione peroxidase (GPX; #MBS456700), catalase (CAT; #MBS728474), 4‐hydroxynonenal (4‐NHE; #MBS7606509), brain natriuretic peptide (BNP; #NBP2‐70011), creatine kinase‐MB (CK‐MB; #ab285231), alcohol dehydrogenase 1 (ADH1; #MBS268092), aldehyde dehydrogenase 2 (ALDH‐2; #MBS726168) and cytochrome P450 2E1 (CYP2E1; #MBS453581).

### 2.8 Quantitative polymerase chain reaction (qPCR)

RNA was isolated using TRIzol reagent and purified via chloroform extraction, isopropanol precipitation and ethanol washing. The RNA was then reverse‐transcribed into cDNA using M‐MLV Reverse Transcriptase. Quantitative PCR was performed using SYBR Select Master Mix on a ViiA 7 Real‐Time PCR System, with Ct values calculated from a standard curve and normalised to GAPDH expression. Primer sequences are listed in Supplementary Table S2.

### 2.9 Western blotting and co‐immunoprecipitation

Protein expression and interactions were analysed using Western blotting and co‐immunoprecipitation (Co‐IP) assays, as previously described.^41^ Samples were homogenised in RIPA buffer and equal amounts of protein were separated by sodium dodecyl‐sulfate polyacrylamide gel electrophoresis (SDS‐PAGE). For Co‐IP, lysates were precleared with normal IgG and protein A/G agarose beads, and then incubated with primary antibody and beads. After washing, immunoprecipitated protein complexes were eluted and analysed by SDS‐PAGE. Antibodies used are listed in Supplementary Table S1.

### 2.10 Measurement of mitochondrial oxygen consumption rate

Mitochondrial respiration was evaluated using established protocols.^42^ Mitochondrial respiration was measured using an XF24 Extracellular Flux Analyzer. Isolated cardiac mitochondria were resuspended in a chilled mitochondrial assay solution containing substrates and inhibitors. Oxygen consumption rates (OCRs) were measured, followed by complete inhibition with antimycin A. Assays were performed in triplicate using pooled mitochondria from 2−4 mice per group. OCRs were measured under basal conditions (coupled state), followed by state 3 respiration (ADP‐stimulated), state 4o (oligomycin‐induced leak state) and state 3u (maximum uncoupled respiration with FCCP). Antimycin A was subsequently added to completely inhibit mitochondrial respiration.

### 2.11 Mitochondrial potential and mitophagy detection

Mitochondrial potential was assessed in cardiomyocytes using the JC‐1 probe (Abcam) following an established protocol.^43^ Cells were incubated with JC‐1, and fluorescence was visualised using a Zeiss microscope. Mitophagy was analysed using the Mito‐Keima assay (Addgene plasmid #56018, MBL Co., Ltd., Tokyo, Japan).^44^ Cells were transfected with a mito‐Keima reporter plasmid (Addgene), and the ratio of fluorescence intensities at 586 and 440 nm indicated mitophagic activity. Images were acquired on a Zeiss microscope, processed using ZEISS ZEN software and quantified using Fiji software and ilastik trainable classification software. The ‘Colour pixel counter’ plugin was used to enumerate pixels of a specific colour within the image. At least 10 cells per group were measured, and results were expressed as a percentage of pixels in the region of interest.

### 2.12 Recombinant adenoviral constructs and cellular transduction

To generate recombinant adenoviruses, murine Pgam5 and Phb2 sequences were cloned into the GV269 vector (CMV‐MCS‐3FLAG‐SV40‐EGFP). Mutant Pgam5 and Phb2 sequences were incorporated into the GV341 vector (Ubi‐MCS‐3FLAG‐SV40‐puromycin) to produce full‐length hemagglutinin (HA)‐tagged wild‐type (WT) Pgam5 (HA‐Pgam5, 1−289 amino acids), HA‐tagged Pgam5 lacking the transmembrane domain (HA‐Pgam5ΔTM, 29−289 amino acids), HA‐tagged Pgam5 lacking the WDXNWD domain (HA‐Pgam5ΔWDXNWD, 1−58 and 63−289 amino acids), HA‐tagged Pgam5 lacking the NXESGE domain (HA‐Pgam5ΔNXESGE, 1−77 and 82−289 amino acids), HA‐tagged Pgam5 lacking the PGAM domain (HA‐Pgam5ΔPGAM5, 1−98 amino acids), full‐length Myc‐tagged WT Phb2 (Myc‐Phb2, 1−299 amino acids), Myc‐tagged Phb2 lacking the N‐terminal domain (Myc‐Phb2ΔN, 50−299 amino acids), Myc‐tagged Phb2 lacking the C‐terminal domain (Myc‐Phb2ΔC, 1−264 amino acids), Myc‐tagged Phb2 lacking the coiled‐coil domain (Myc‐Phb2ΔCC, 1−190 and 264−299 amino acids) and Myc‐tagged Phb2 lacking the prohibition (PHB) domain (Myc‐Phb2ΔPHB, 1−68 and 185−299 amino acids). All adenoviral vectors were constructed by GeneChem (Shanghai, China). HL‐1 cardiomyocytes were transduced with the respective adenoviruses at a multiplicity of infection (MOI) of 10, with no observable cytotoxicity. Additionally, neonatal murine cardiomyocytes were transfected with Pgam5 siRNA (sc‐152184, Santa Cruz Biotechnology, Inc.), NDUFA5 siRNA (sc‐149866, Santa Cruz Biotechnology, Inc.), or UQCRC1 siRNA (sc‐72155, Santa Cruz Biotechnology, Inc.), as previously described.^42^

### 2.13 In silico protein‐ligand docking

In silico docking simulations were performed using AutoDock Vina to investigate the binding modalities between Pgam5 and Phb2. The 3D protein structures were obtained from the RCSB Protein Data Bank. AutoDockTools prepared the input files, and the ligand (Pgam5) was processed to merge non‐polar hydrogen atoms and define rotatable bonds. The exhaustiveness parameter was set to 16 for a thorough conformational search. The top‐scoring pose was selected based on the Vina docking score and visually analysed using PyMoL software.

### 2.14 Statistical analyses

Data analysis was conducted using GraphPad Prism 8, with results presented as mean ± SEM. Outliers were identified using Grubbs' test, and the Shapiro‐Wilk test was employed to determine data normality. Statistical significance (p < .05) was evaluated using unpaired t‐tests for two‐group comparisons and one‐way analysis of variance (ANOVA) with Tukey's correction for multiple group comparisons, as appropriate.

## 3 RESULTS

### 3.1 *Pgam5* knockout attenuates alcohol‐induced cardiac histopathology and heart dysfunction in male mice

**FIGURE 1:**
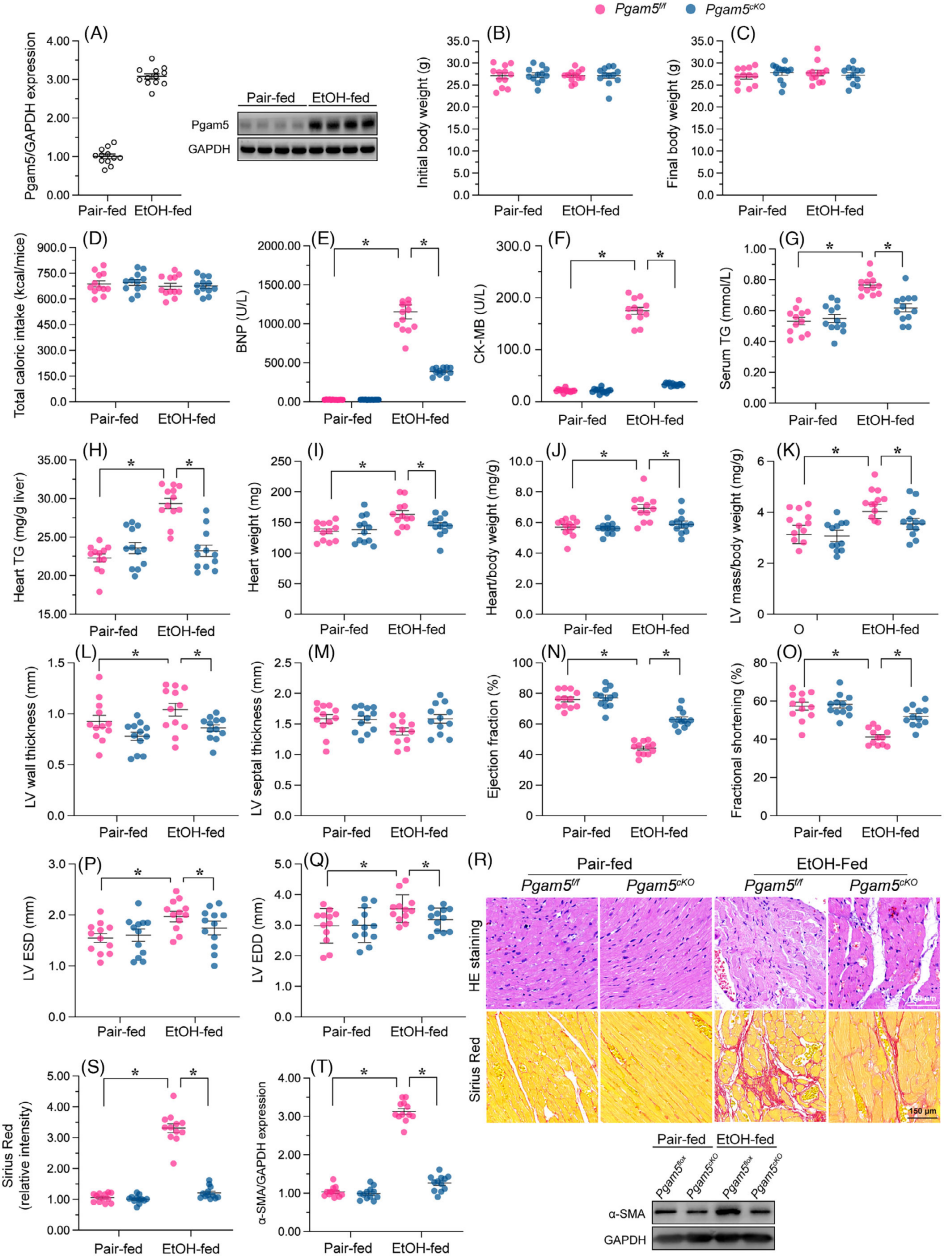
*Pgam5* KO improves heart histopathology and function in a male mouse model of ACM. Male WT, *Pgam5* knockout (*Pgam5^cKO^*) and *Pgam5^f/f^* mice were pair‐fed a liquid control or a 5% ethanol‐containing diet for 8 weeks. (A) Western blot analysis of myocardial Pgam5 protein expression in male WT mice. (B–K) Initial body weight (B), final body weight (C), Caloric intake (D), serum BNP (E), serum CK‐MB (F), serum TG (G), heart TG (H), heart weight (I), heart/body weight (J) and left ventricular (LV) mass/body weight (K) measurements in male *Pgam5^cKO^* and male *Pgam5^f/f^* mice. (L–Q) Echocardiography was used to analyse the heart function. LV ESD, left ventricular end systolic diameter; LV EDD, left ventricular end diastolic diameter. (R) Histopathological analysis (H&E staining) of heart tissue from male *Pgam5^cKO^* and male *Pgam5^f/f^* mice. (S) Analysis of myocardial fibrosis (picro‐Sirius red staining) in male *Pgam5^cKO^* and male *Pgam5^f/f^* mice. (T) Western blot analysis of α‐SMA protein expression in heart tissues. Experiments were repeated at least three times and the data are shown as mean ± SEM. Experiments were repeated at least three times and the data are shown as mean ± SEM (N = 12 mice per group). *P < .05.

To investigate Pgam5's role in alcohol‐induced myocardial injury, WT mice were fed a control or 5% ethanol‐containing diet for 8 weeks. Western blots showed that Pgam5 expression was upregulated by EtOH treatment (Figure 1A). To evaluate whether a causal‐effect existed between Pgam5 overexpression and myocardial injury, male cardiomyocyte‐specific *Pgam5* knockout (*Pgam5^cKO^*) and *Pgam5^f/f^* control mice were tested. A schematic of the construction of *Pgam5^cKO^* mice was shown in Supplemental Figure S1. Neither EtOH feeding nor *Pgam5* KO influenced body weight (Figure 1B and 1C) or caloric intake (Figure 1D) in male mice. However, ethanol intake elevated the concentrations of BNP and CK‐MB in male *Pgam5^f/f^* mice, and these indices were partially reduced in male *Pgam5^cKO^* mice (Figure 1E and 1F). Besides, upon EtOH treatment, both serum and heart TG concentrations were markedly increased in male *Pgam5^f/f^* mice, but less so in male *Pgam5^cKO^* mice (Figure 1G and 1H). Additionally, EtOH treatment in male *Pgam5^f/f^* mice led to an increase in heart weight, heart/body weight ratio and left ventricular (LV) mass/body weight ratio (Figure 1I–1K). However, these alterations were mitigated in male *Pgam5^cKO^* mice. Echocardiography analysis revealed that EtOH impaired heart function in male *Pgam5^f/f^* mice, but this impairment was normalised in male *Pgam5^cKO^* mice (Figure 1L–Q).

Histological examination revealed that ethanol exposure induced a spectrum of detrimental alterations in myocardial fibre architecture, including disarrayed fibre alignment, swelling, fragmentation and aberrant twisting, in the hearts of male *Pgam5^f/f^* mice, but not in those of male *Pgam5^cKO^* mice (Figure 1R). Based on Sirius red staining, after EtOH treatment, mild cardiac fibrosis was also noted in male *Pgam5^f/f^* mice (Figure 1R and 1S) but not in male *Pgam5^cKO^* mice. These data indicated that *Pgam5* KO protects the heart against alcohol‐caused structural and functional abnormalities in male mice. Western blot analysis demonstrated a significant upregulation of a myocardial fibrosis marker in response to ethanol exposure in male *Pgam5^f/f^* mice compared to male *Pgam5^cKO^* mice (Figure 1T).

Previous studies have reported that women have a greater risk of developing alcohol‐related diseases than men.^35^ In accordance with these findings, we found that female mice have higher levels of Pgam5 (Supplemental Figure S2A–B) and BNP/CK‐MB (Supplemental Figure S2C–D) after exposure to EtOH when compared with that in male mice. Besides, less ADH/ALDH concentration and increased CYP2E1 activity in heart tissues were also observed in female mice upon EtOH treatment when compared with that in male mice (Supplemental Figure S2E–G). Histological analysis using HE staining (Supplemental Figure S2H) and Sirius red staining (Supplemental Figure S2I–J) also confirmed that female mice have more obvious structural alterations in heart tissue upon EtOH exposure relative to that in male mice. Interestingly, although Pgam5 expression was also upregulated in heart tissues from female mice, *Pgam5* KO cannot significantly interrupted EtOH‐caused abnormalities of heart function (Supplemental Figure S2C–G) and structure (Supplemental Figure S2H–J) in female mice, suggesting that other signalling transduction pathways in addition to Pgam5, is induced by alcohol in female mice, thereby contributing to augmented heart injury compared to male mice. Based on this, male mice were used in our studies to explore the molecular basis of Pgam5 in alcoholic heart disease.

### 3.2 *Pgam5* KO reduces alcohol‐induced myocardial inflammation, oxidative stress and metabolic dysfunction in male mice

**FIGURE 2:**
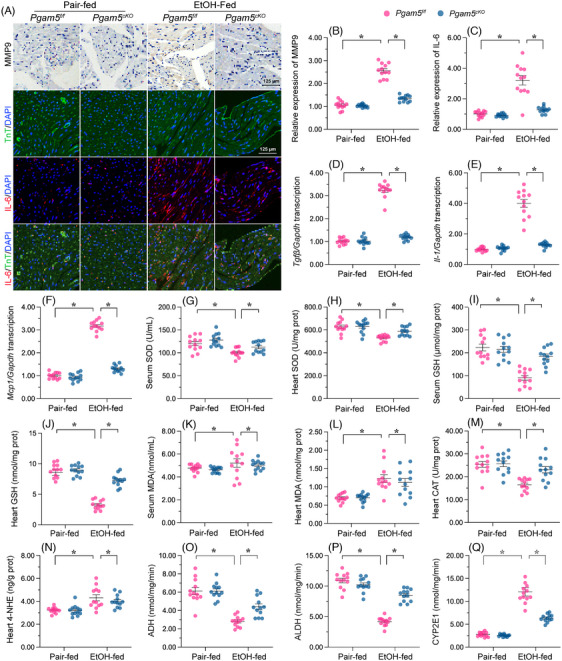
*Pgam5* KO attenuates EtOH‐related inflammation and lipid peroxidation, and normalises metabolic enzyme levels in male mouse heart tissue. (A–C) Representative images of double immunohistochemistry of MMP9 and immunofluorescence of IL‐6 in heart tissues from male *Pgam5^cKO^* and male *Pgam5^f/f^* mice. (D–F) Transcriptional analysis of *Tgfβ*, *Il‐1 and Mcp1* expression in heart tissues from male *Pgam5^cKO^* and male *Pgam5^f/f^* mice. (G–L) ELISA‐based analysis of serum and heart levels of SOD, GSH and MDA in male *Pgam5^cKO^* and male *Pgam5^f/f^* mice. (M–N) ELISA‐based analysis of CAT and 4‐NHE levels in heart tissues from male *Pgam5^cKO^* and male *Pgam5^f/f^* mice. (O–Q) ELISA‐based analysis of ADH, ALDH and CYP2E1 levels in heart tissues from male *Pgam5^cKO^* and male *Pgam5^f/f^* mice. Experiments were repeated at least three times and the data are shown as mean ± SEM (N = 12 mice per group). *p < .05.

The progression of ACM is thought to involve a complex interplay of factors, including inflammatory responses, redox imbalance stress and disrupted alcohol metabolism.^45^, ^46^, ^47^, ^48^ Immunohistochemistry and immunofluorescence assays showed that after EtOH treatment, myocardial expression of MMP9 and IL‐6 was elevated in male *Pgam5^f/f^* mice and markedly downregulated in male *Pgam5^cKO^* mice (Figure 2A–2C). In accordance with these findings, the mRNA expression of *Tgfβ*, *Il‐1* and *Mcp1* was increased by EtOH treatment in male *Pgam5^f/f^* mice, and reduced instead to near‐normal levels after *Pgam5* knockout (Figure 2D–2F). Oxidative stress in cardiomyocytes is featured by decreased levels of antioxidative enzymes and increased levels of lipid peroxidation products. ELISA demonstrated that serum and heart concentrations of SOD and GSH were reduced upon EtOH administration, an effect paralleled by an increase in the respective concentrations of MDA (Figure 2G–2L). Besides, heart CAT content was downregulated, whereas 4‐NHE content was upregulated, in male *Pgam5^f/f^* mice (Figure 2 M and 2N) but not in male *Pgam5^cKO^* mice. In turn, the activities of ADH and ALDH, two enzymes involved in myocardial metabolism of alcohol, were significantly decreased (Figure 2O and 2P), in parallel with increased activity of CYP2E1 (Figure 2Q). These findings indicate that loss of Pgam5 prevents a decline in ADH and ALDH activities (Figure 2O and 2P) and maintains the concentration of CYP2E1 (Figure 2Q) in the male mouse model of ACM.

### 3.3 *Pgam5* depletion protects mitochondrial function in alcohol‐exposed male mouse cardiomyocytes

**FIGURE 3:**
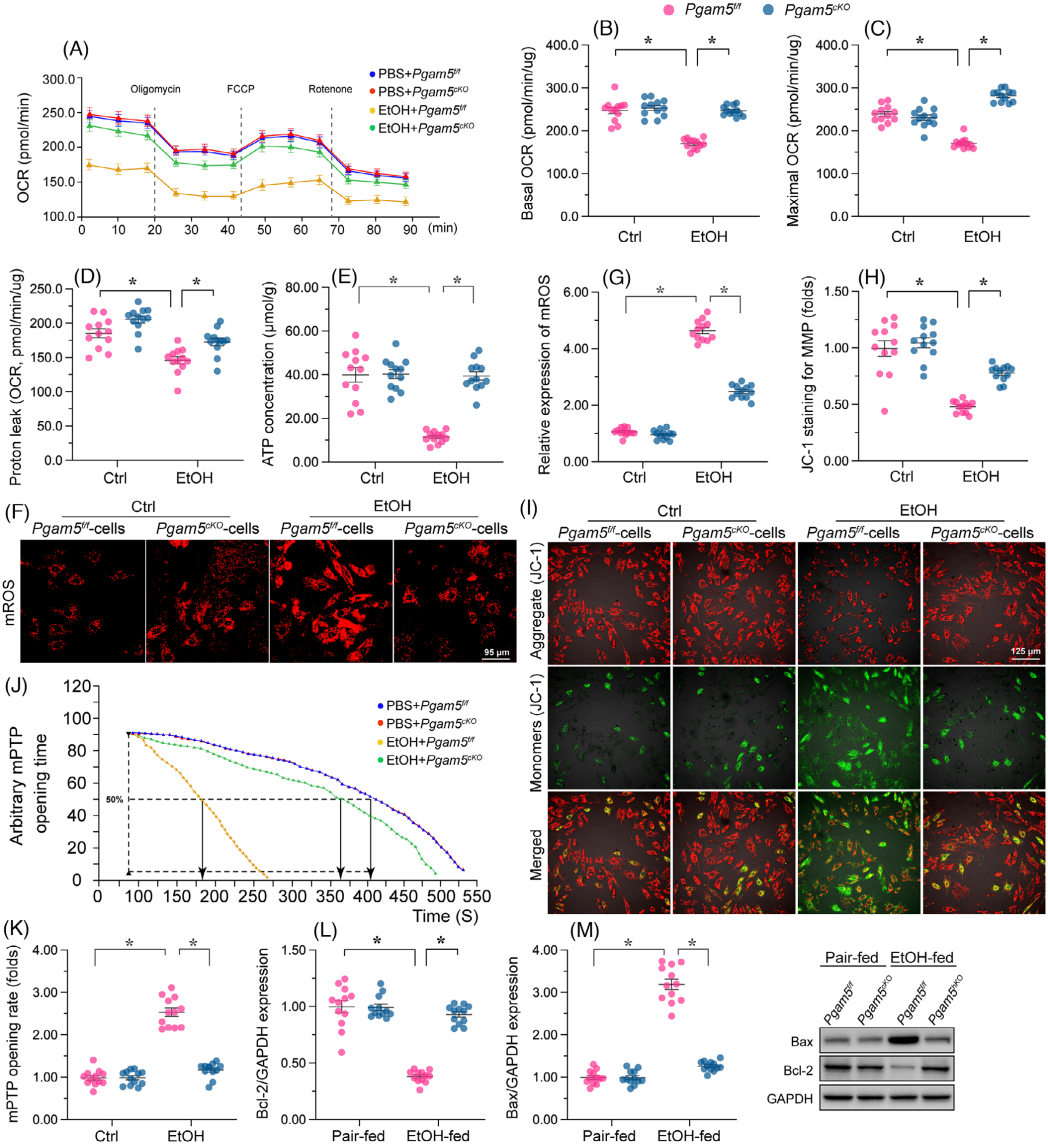
*Pgam5* depletion protects mitochondrial function in ACM. Neonatal cardiomyocytes were isolated from male *Pgam5^f/f^* and *Pgam5^cKO^* mice and then treated with ethanol (100 mM) for 48 h. (A–D) Analysis of mitochondrial respiration (OCR assay) in male mouse neonatal cardiomyocytes. (E) The changes of ATP were detected via ELISA assay in male mouse neonatal cardiomyocytes. (F, G) Representative images depicting changes in mitochondrial ROS production in male mouse neonatal cardiomyocyte loaded with the ROS‐sensitive MitoSOX Red probe. (H, I) Analysis of changes in mitochondrial membrane potential in male mouse neonatal cardiomyocytes loaded with the potentiometric indicator JC‐1. (J, K) TMRE‐based determination of mPTP opening rate in male mouse neonatal cardiomyocytes. (L–N) Western blot analysis of Bax and Bcl‐2 expression in heart tissues from male *Pgam5^cKO^* and male *Pgam5^f/f^* mice. Experiments were repeated at least three times and the data are shown as mean ± SEM (N = 12 mice or three independent cell isolations per group). *p < .05.

Mitochondrial damage is a hallmark of ACM. Considering Pgam5's key role in regulating mitochondrial dynamics, we investigated whether Pgam5 deletion mitigates mitochondrial dysfunction in male mice with ACM. To evaluate this hypothesis, mitochondrial metabolism was first determined by measuring OCR. Baseline mitochondria respiration and maximal OCR were both impaired by EtOH in neonatal cardiomyocytes isolated from male *Pgam5^f/f^* mice (Figure 3A–D), an effect accompanied by increased proton leak and ATP turnover (Figure 3E). Notably, *Pgam5* deletion was able to maintain baseline and maximal OCR (Figure 3A–3D), as well as to reduce proton leak and ATP turnover under EtOH stress (Figure 3E). Decreased mitochondrial bioenergetics is closely associated with increased generation of ROS and decreased mitochondrial potential. MitoSOX Red assays showed that EtOH promoted mROS generation in cardiomyocytes from male *Pgam5^f/f^* but not from male *Pgam5^cKO^* mice (Figure 3F and 3G). Similarly, EtOH treatment led to dissipation of mitochondrial potential in cardiomyocytes from male *Pgam5^f/f^* but not from male *Pgam5^cKO^* mice (Figure 3H and 3I). Due to abnormal mitochondrial function, mitochondria‐mediated cell apoptosis may be expectably induced during ACM. The mPTP opening time is considered a reliable marker of mitochondria‐mediated cell death. After exposure to EtOH, the opening time of the mPTP was prolonged in male *Pgam5^f/f^* cardiomyocytes (Figure 3J and 3K). Parallelly, suggesting activation of mitochondria‐induced apoptosis, the expression of Bax was increased, whereas Bcl‐2 levels were reduced in those cells (Figure 3L and 3M ). In contrast, a shortened mean mPTP opening time (Figure 3J and 3K) and a normalised Bcl‐2/Bax ratio (Figure 3L and 3M ) were observed in male *Pgam5*‐deficient cardiomyocytes. These data indicated that abnormal Pgam5 expression contributes to EtOH‐induced mitochondrial dysfunction in mouse cardiomyocytes.

To further investigate whether *Pgam5* KO prevents alcohol‐induced cardiomyocyte death by preserving mitochondrial function, we selectively targeted two genes associated with mitochondrial function: NDUFA5, which encodes mitochondrial respiration complex I, and UQCRC1, which maintains the function of mitochondrial respiration complex III. In neonatal cells isolated from male WT mice, NDUFA1/siRNA or cytochrome c/siRNA were transfected before *Pgam5* KO. In cells transfected with either NDUFA1/siRNA or UQCRC1 /siRNA, *Pgam5* KO failed to sustain cell viability (Supplemental Figure S3), suggesting that *Pgam5* KO protects mitochondrial function, subsequently safeguarding cardiomyocyte survival.

### 3.4 *Pgam5* depletion normalises MQC in ACM

**FIGURE 4:**
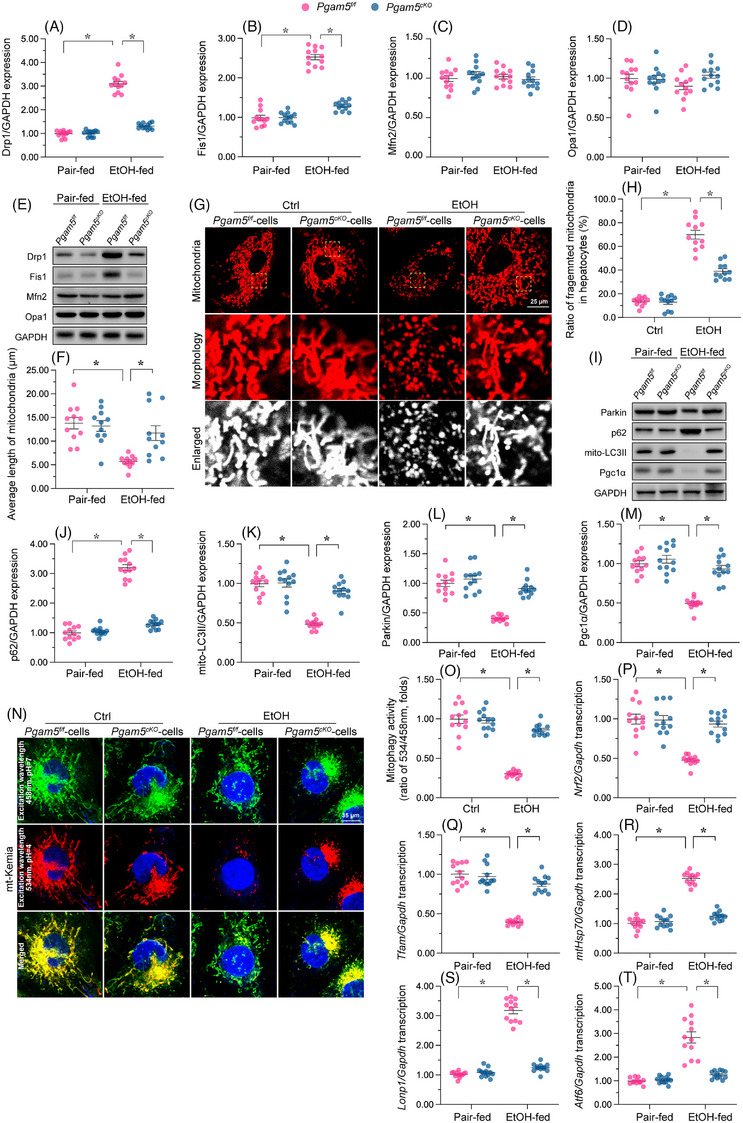
*Pgam5* depletion normalises MQC in alcohol‐challenged male mouse. (A–E) Western blot analysis of mitochondrial fission‐related (Drp1, Fis1) and fusion‐related (Mfn2, Opa1) proteins in heart tissues from male *Pgam5^cKO^* and male *Pgam5^f/f^* mice. (F–H) Representative images of Tom20 immunofluorescence, used to analyse mitochondrial morphology in male mouse neonatal cardiomyocytes, and quantification of fragmented mitochondria and average mitochondrial length. (I–M) Western blot analysis of mitochondrial LC3II (mito‐LC3II), p62, Parkin and Pgc1α in heart tissues from male *Pgam5^cKO^* and male *Pgam5^f/f^* mice. (N, O) Representative images of cardiomyocytes transfected with the mitophagy indicator mito‐Keima. (P–T) Transcriptional analysis of *Tfam, Nrf2, Lonp1*, *mtHsp70* and *Atf6* in heart tissues from male *Pgam5^cKO^* and male *Pgam5^f/f^* mice. Experiments were repeated at least three times and the data are shown as mean ± SEM (N = 12 mice or three independent cell isolations per group). *p < .05.

Under stress conditions, proper functioning of the MQC is critical to sustain mitochondrial integrity and function via coordinated regulation of fission/fusion events, mitophagic activity and mitochondrial regeneration.^49^, ^50^ Given the beneficial impact of *Pgam5* deletion on heart mitochondrial performance in male mice during ACM in vivo and during EtOH exposure in vitro, we next asked whether *Pgam5* ablation in cardiomyocytes attenuates alcohol‐induced mitochondrial dysfunction by activating the MQC system. Western blot assays on heart tissues illustrated that the expression of fission‐related regulators, namely Drp1 and Fis1, was rapidly elevated in response to alcohol treatment, and these changes were abrogated by *Pgam5* KO (Figure 4A–4E). Interestingly, neither alcohol nor *Pgam5* KO impacted the expression of the fusion‐related regulators Mfn2 and Opa1 (Figure S4A–4E). Those data suggested that fission, rather than fusion, is controlled by Pgam5 in alcohol‐exposed male mouse hearts. Next, Tom20 immunofluorescence was used to analyse the structure of mitochondria in neonatal male mouse cardiomyocytes. As seen in Figure 4F–4H, upon EtOH exposure the percentage of fragmented, round mitochondria reached 71% in male *Pgam5^f/f^* cardiomyocytes but only 40% in male *Pgam5^cKO^* cardiomyocytes. Accordingly, upon EtOH stress, the average length of mitochondria was significantly shortened in cardiomyocytes from male *Pgam5^f/f^* mice, but maintained instead at near‐physiological values in male *Pgam5^cKO^* cardiomyocytes (Figure 4F–4H).

Accompanying enhanced mitochondrial fission, mitophagy was significantly inhibited by alcohol treatment in the male *Pgam5^f/f^* mouse heart, as shown by reduced levels of Parkin and LC3, as well as increased levels of p62 (Figure 4I–4M ). In contrast, these changes were largely normalised after *Pgam5* KO (Figure 4I–4M ). Accordingly, in vitro experiments using mito‐Keima‐transfected neonatal cardiomyocytes revealed that ethanol exposure significantly suppressed mitophagic activity in *Pgam5^f/f^* cardiomyocytes, but not in *Pgam5*‐deficient cells (Figure 4N–4O).

Mitochondrial biogenesis, a regenerative process that serves to replace old, dysfunctional mitochondria, is mainly regulated by Pgc1α and several co‐transcriptional factors. We found that following EtOH feeding, the levels of Pgc1α protein was inhibited (Figure 4I and 4M ), while the mRNA levels of both *Tfam* and *Nrf2* were reduced, in the hearts of male *Pgam5^f/f^* mice (Figure 4P and 4Q). In contrast, both Pgc1α expression (Figure 4I and 4M ) and *Tfam* and *Nrf2* transcription (Figure 4P and 4Q) were largely restored in heart tissue from male *Pgam5^cKO^* mice.

We finally examined the mtUPR, a mitochondria‐controlled nuclear transcriptional process which regulates, similar to the endoplasmic reticulum‐associated UPR, the transcription of stress‐related genes to attenuate abnormal protein folding.^51^ We conducted qPCR assays which showed that in response to EtOH feeding, the mRNA levels of mtUPR‐related factors in heart tissue were obviously increased in male *Pgam5^f/f^* mice, but remained instead at baseline levels in male *Pgam5^cKO^* animals (Figure 4R–4T). In sum, these results indicated that in the setting of ACM, *Pgam5* deficiency significantly sustains MQC stability in heart by inhibiting mitochondrial fission and normalising mitophagy, mitochondrial biogenesis and the mtUPR.

### 3.5 EtOH impairs MQC through inducing Phb2 dephosphorylation at Ser91

**FIGURE 5:**
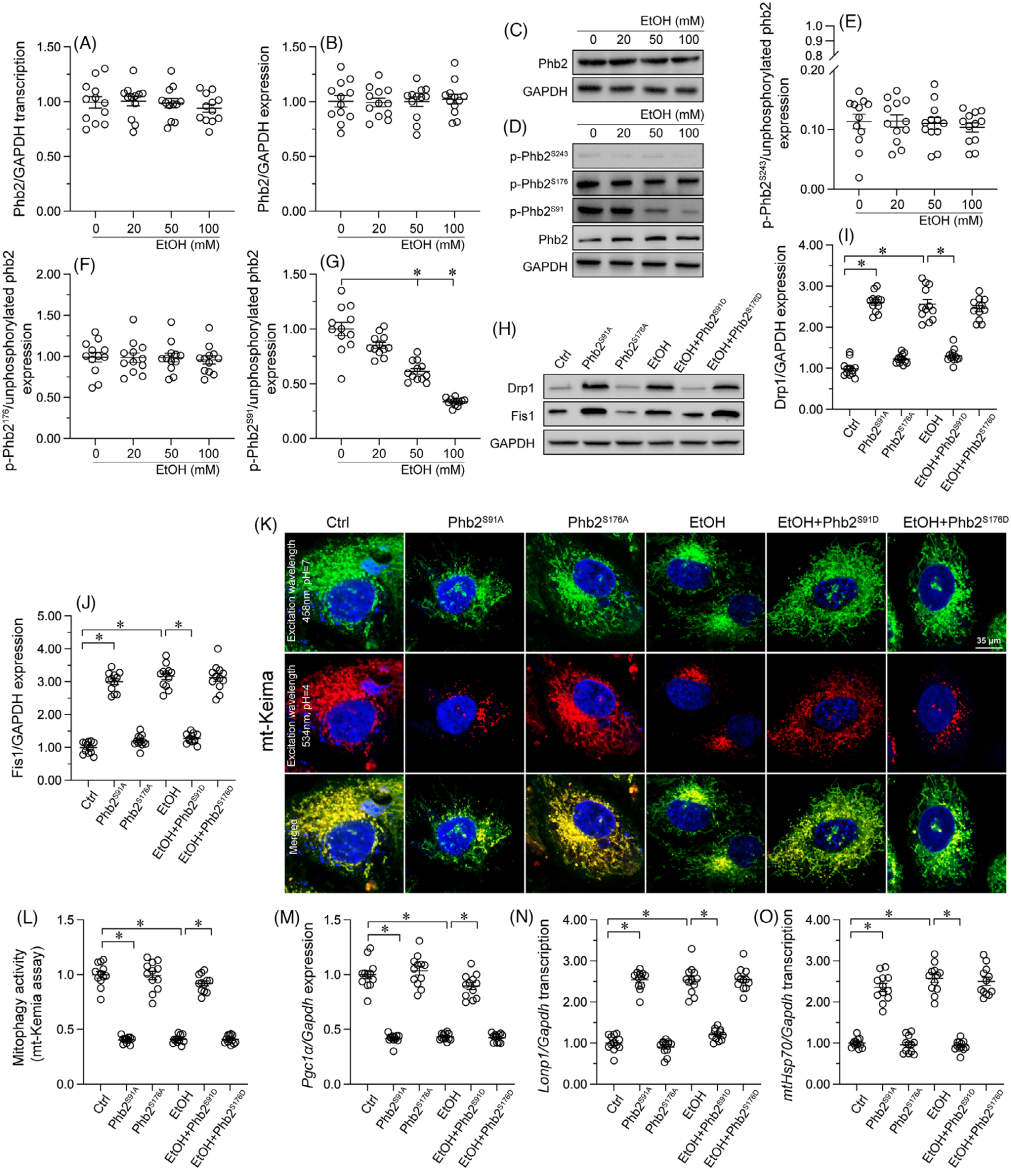
EtOH impairs MQC by inducing Phb2 dephosphorylation at Ser91. HL‐1 cardiomyocytes were alternatively transduced with two phospho‐mimetic (Phb2^S91D^ and Phb2^S176D^) and two phospho‐defective (Phb2^S91A^ and Phb2^S176A^) mutants of Phb2 before EtOH treatment. (A) Transcriptional analysis of *Phb2* expression in heart tissues from male WT mice treated with or without EtOH. (B, C) Western blots analysis of myocardial Phb2 expression in male WT mice‐derived cardiomyocytes. (D–G) Western blots analysis of Phb2 phosphorylation in heart form male WT mice‐derived cardiomyocytes. (H–J) Western blot analysis of Drp1 in HL‐1 cells alternatively transduced with Phb2^S91D^, Phb2^S176D^, Phb2^S91A^ and Phb2^S176A^. (K, L) Representative images of mito‐Keima‐transduced HL‐1 cells expressing Phb2^S91D^, Phb2^S176D^, Phb2^S91A^ or Phb2^S176A^. (M–O) Transcriptional analysis of *Pgc1α, Lonp1* and *mtHsp70* expression in HL‐1 cells transduced with Phb2^S91D^, Phb2^S176D^, Phb2^S91A^ or Phb2^S176A^. Experiments were repeated at least three times and the data are shown as mean ± SEM (N = three independent cell isolations per group). *p < .05.

Although our previous studies and other researchers’ work identified Phb2 as a novel regulator of MQC in various disease models,^52^, ^53^ this phenomenon has not yet been confirmed in male mice during ACM. To understand the mechanisms responsible for MQC dysfunction under EtOH‐induced stress, the transcription and expression of Phb2 were determined in heart tissue from male ACM mice. Interestingly, EtOH feeding did not affect myocardial *Phb2* transcription (Figure 5A) or expression (Figure 5B and 5C) in WT mice. Building upon recent findings that post‐translational phosphorylation enhances Phb2 function,^54^, ^55^ we investigated the phosphorylation status of Phb2 in EtOH‐treated neonatal cardiomyocytes derived from male mice. Western blot analyses failed to detect measurable expression of p‐Phb2^S243^, but revealed instead abundant levels of p‐Phb2^S176^ and p‐Phb2^S91^ at baseline (Figure 5D–5G). Interestingly, EtOH treatment had no influence on p‐Phb2^S176^, but dose‐dependently reduced the expression of p‐Phb2^S91^ (Figure 5D–5G), suggesting that Phb2 dephosphorylation at S91 is a pathogenic modification induced by ACM.

To investigate the potential influence of decreased p‐Phb2^S91^ levels in EtOH‐related MQC dysfunction, two Ser91 phospho‐mimetic (Phb2^S91D^ and Phb2^S176D^) as well as two Ser91 phospho‐defective (Phb2^S91A^ and Phb2^S176A^) mutants of Phb2 were generated and then transduced into HL‐1 cardiomyocytes before EtOH treatment. Western blotting illuminated that the levels of Drp1/Fis1 were enhanced by either EtOH or Phb2^S91A^ transduction. However, these changes were attenuated upon forced expression of Phb2^S91D^ (Figure 5H–5J). In turn, mitophagic activity was significantly repressed by either EtOH or Phb2^S91A^ expression, but sustained instead after EtOH treatment in HL‐1 cardiomyocytes transduced with Phb2^S91D^ (Figure 5K and 5L). Similarly, the transcription of *Pgc1α* was downregulated, whereas mtUPR‐related markers were transcriptionally upregulated in response to EtOH stress or Phb2^S91A^ transduction, but restored instead upon Phb2^S91D^ transduction (Figure 5M–5O ). Lastly, regardless of EtOH treatment, transduction with Phb2^S176D^ had no protective actions on mitochondrial fission (Figure 5H–5J), mitophagy (Figure 5K and 5L), biogenesis (Figure 5M) or mtUPR (Figure 5N and 5O) in the presence of EtOH challenge, implying an irrelevant role of p‐Phb2^S176^ in MQC dysfunction during alcohol attack. Overall, these data suggested that alcohol exposure disrupts MQC in cardiomyocytes through inducing Phb2 dephosphorylation at Ser91.

### 3.6 Pgam5 interacts with Phb2 and induces Phb2 dephosphorylation

**FIGURE 6:**
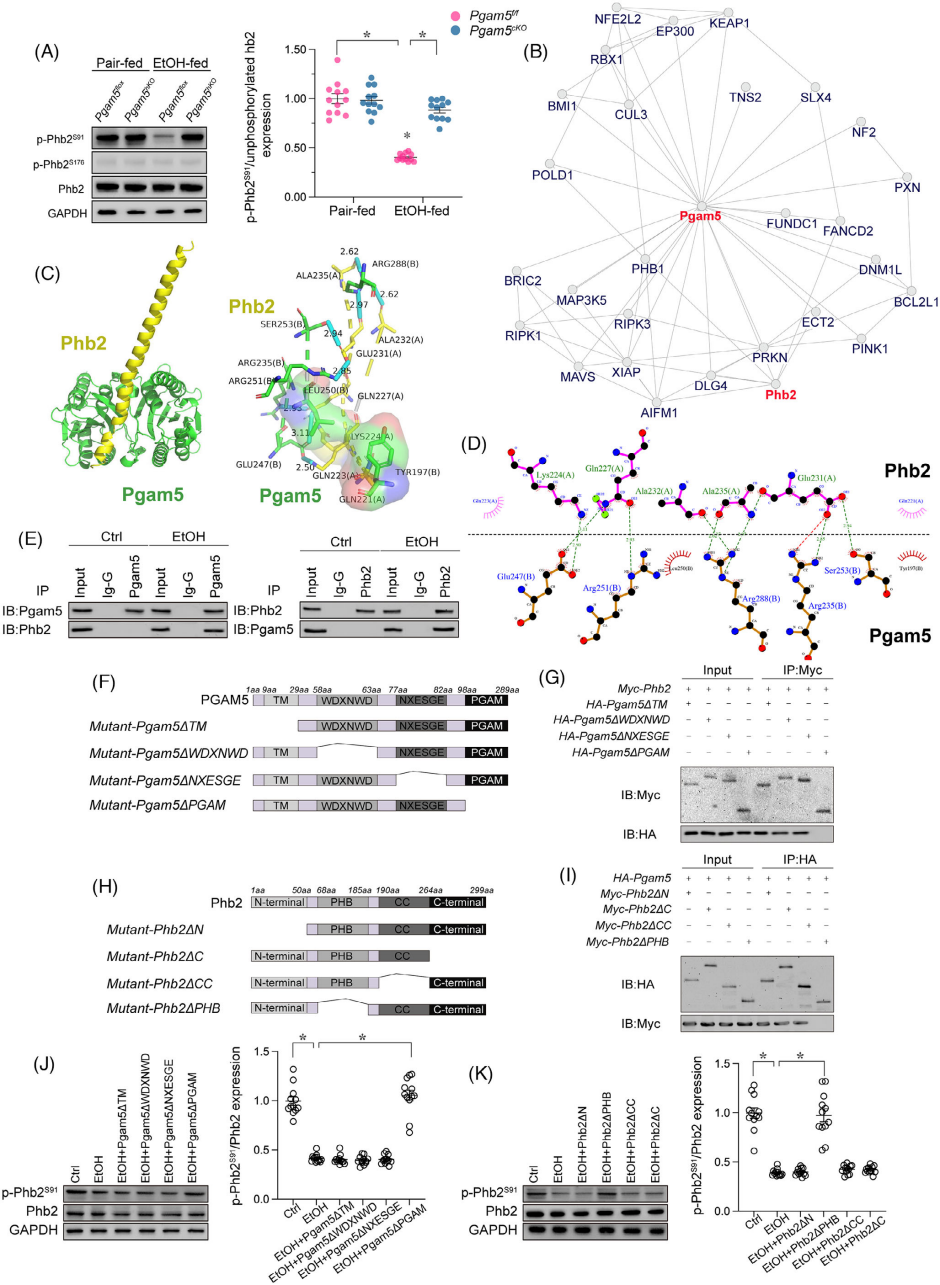
EtOH exposure induces Pgam5‐mediated Phb2 dephosphorylation. (A) Western blot analysis of Phb2 phosphorylation in heart tissues from male *Pgam5^f/f^* and male *Pgam5^cKO^* mice. (B) Predicted interaction between Pgam5 and Phb2 based on analysis in the inBio Discover database. (C) Computational docking model of the interaction between Pgam5 and Phb2. (D) Schematic depiction of key interacting sites in Pgam5 and Phb2. (E) Results of Co‐IP assays indicating interaction between Pgam5 and Phb2 in male mouse cardiomyocytes challenged with EtOH. (F) Mapping of Pgam5 domains. (G) Co‐IP analysis of Pgam5‐Phb2 binding in HL‐1 cells transduced with Pgam5 domain mutants lacking the TM domain (Pgam5ΔTM), the WDXNWD domain (Pgam5ΔWDXNWD), the NXESGE domain (Pgam5ΔNXESGE) or the PGAM domain (Pgam5ΔPGAM). (H) Mapping of Phb2 domains. (I) Co‐IP analysis of Pgam5‐Phb2 binding in HL‐1 cells transduced with Phb2 domain mutants lacking the N‐terminal domain (Phb2ΔN), the C‐terminal domain (Phb2ΔC), the coiled‐coil domain (Phb2ΔCC) or the PHB domain (Phb2ΔPHB). (J) Western blot analysis of Phb2 phosphorylation in HL‐1 cells expressing mutant Pgam5 domains. (K) Western blot analysis in HL‐1 cells expressing mutant Phb2 domains. Experiments were repeated at least three times and the data are shown as mean ± SEM (N = three independent cell isolations per group). *p < .05.

Considering that cardiomyocyte‐specific deletion of *Pgam5* protected the heart against alcoholic injury by stabilising MQC, we therefore questioned whether Phb2 dephosphorylation at Ser91 results from Pgam5 activity. Confirming this hypothesis, western blot analysis showed that EtOH‐mediated Phb2 dephosphorylation in heart tissue occurred in male *Pgam5^f/f^* but not in male *Pgam5^cKO^* mice (Figure 6A). Structural analysis of protein‐protein interaction in the inBio Discover platform (https://inbio‐discover.com) predicted potential binding between Pgam5 and Phb2 (Figure 6B). Such interaction was further confirmed through docking simulations, which revealed also key interactive sites between these two proteins (Figure 6C and 6D). Meanwhile, Co‐IP experiments revealed increased interaction between Pgam5 and Phb2 in EtOH‐treated, compared to untreated, male mouse cardiomyocytes (Figure 6E).

After asserting the preferential binding between Pgam5 and Phb2 in EtOH‐exposed male mouse neonatal cardiomyocytes, we sought to unmask the structural determinants of such interaction. Co‐IP analyses in HL‐1 cardiomyocytes showed that transduction of a Pgam5 mutant lacking the PGAM domain (Pgam5ΔPGAM), but not of mutant Pgam5 vectors lacking the TM domain (Pgam5ΔTM), the WDXNWD domain (Pgam5ΔWDXNWD) or the NXESGE domain (Pgam5ΔNXESGE), prevented Pgam5/Phb2 interaction upon EtOH treatment (Figure 6F and 6G). Similarly, in the presence of EtOH, Pgam5/Phb2 binding was inhibited in cells transduced with a Phb2 mutant lacking the PHB domain (Phb2ΔPHB), but not in those expressing Phb2 mutants lacking the N‐terminal domain (Phb2ΔN), the C‐terminal domain (Phb2ΔC) or the coiled‐coil domain (Phb2ΔCC) (Figure 6H and 6I). The above findings highlighted that the PGAM domain of Pgam5 and the PHB domain of Phb2 are involved in their interplay induced by EtOH. Lastly, to confirm the specificity of the above domains in Pgam5‐mediated dephosphorylation of Phb2, we conducted western blot analyses which showed that EtOH‐induced Phb2 dephosphorylation was blunted in HL‐1 cardiomyocytes transduced with Pgam5ΔPGAM, but remained largely unaffected in cells expressing Pgam5ΔTM, Pgam5ΔWDXNWD or Pgam5ΔNXESGE (Figure 6J). Similarly, after EtOH exposure, Phb2 dephosphorylation was abrogated in HL‐1 cells transduced with Phb2ΔPHB, but not in those expressing Phb2ΔN, Phb2ΔC or Phb2ΔCC (Figure 6K). These findings indicate that in the presence of EtOH, Pgam5 dephosphorylates Phb2 via binding through their respective PGAM and PHB domains.

### 3.7 Disruption of Pgam5/Phb2 binding reduces EtOH‐induced mitochondrial damage in cardiomyocytes

To confirm whether the Pgam5/Phb2 interaction accounts for decreased mitochondrial function in EtOH‐treated HL‐1 cardiomyocytes, Pgam5ΔPGAM and Phb2ΔPHB constructs were alternatively transduced into HL‐1 cardiomyocytes before EtOH treatment. Compared to control cells, mitochondrial ATP production was significantly restored (Supplemental Figure S4A), mitochondrial potential was maintained (Supplemental Figure S4B–C) and mROS production was inhibited (Supplemental Figure S4D–E) in EtOH‐exposed HL‐1 cells previously transduced with either Pgam5ΔPGAM or Phb2ΔPHB. Besides, upon EtOH stress, the mPTP opening rate was restored to baseline levels in those cells (Supplemental Figure S4F). These results indicate that disruption of Pgam5/Phb2 interaction sustains mitochondrial function in EtOH‐challenged cardiomyocytes.

### 3.8 Inhibition of Pgam5/Phb2 binding attenuates EtOH‐mediated cardiomyocyte injury

To investigate whether EtOH‐mediated cardiomyocyte damage is associated with enhanced Pgam5/Phb2 interaction, cardiomyocyte function was evaluated again in Pgam5ΔPGAM‐ and Phb2ΔPHB‐transduced HL‐1 cardiomyocytes. Cell viability was significantly impaired in control cells upon EtOH attack, and this alteration was alleviated by transduced of Pgam5ΔPGAM or Phb2ΔPHB (Supplemental Figure S5A). Besides, EtOH‐induced pro‐inflammatory reaction was inhibited after transduced with the above Pgam5 and Phb2 mutants (Supplemental Figure S5B–D). In addition, both manoeuvres restored ADH/ALDH levels and inhibited CYP2E1 upregulation in EtOH‐treated HL‐1 cardiomyocytes (Supplemental Figure S5E–G). Finally, expression of Pgam5ΔPGAM or Phb2ΔPHB in EtOH‐challenged HL‐1 cells rescued their antioxidant defences (Supplemental Figure S5H–J). These data indicated that disruption of Pgam5/Phb2 binding reverses cardiomyocyte dysfunction induced by EtOH.

#### 3.8.1 Mice genetically modified to express a phosphor‐mimetic Phb2 mutant at *serine* 91 exhibit resistance to the development of ACM

**FIGURE 7:**
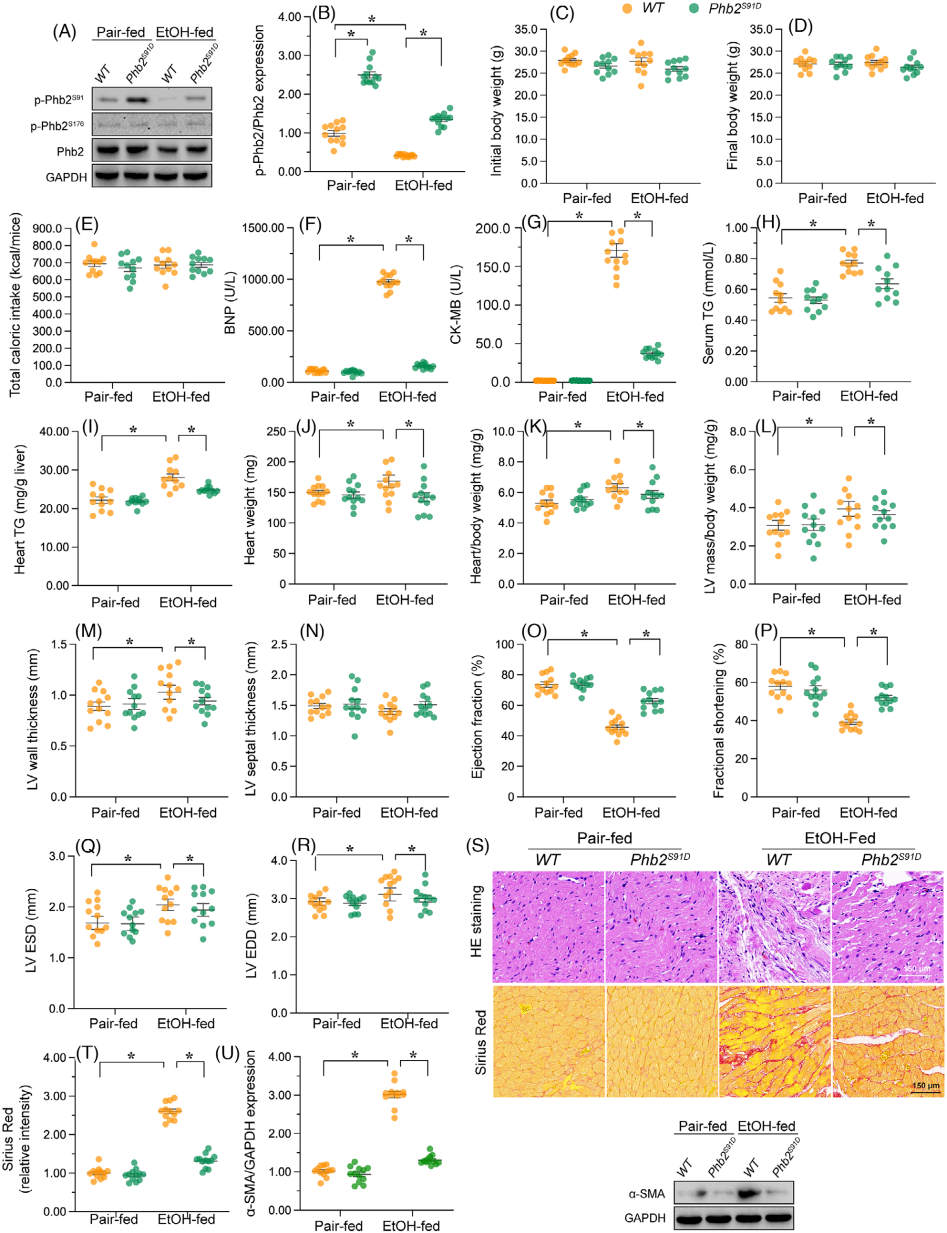
Transgenic mice expressing a Ser91 phospho‐mimetic Phb2 mutant are resistant to ACM. Male wild‐type (WT) and male homozygous *Phb2^S91D^* mice (*Phb2^D/D^*) were pair‐fed a liquid control or a 5% ethanol‐containing diet for 8 weeks. (A, B) Western blot analysis of p‐Phb2^S91^ and total PHB2 levels in heart tissues from male WT and male *Phb2^D/D^* mice. (C‐L) Initial body (C), final body (D), caloric intake (E), BNP (F), serum CK‐MB (G), serum TG (H), heart TG (I), heart weight (J), heart/body weight (K) and left ventricular (LV) mass/body weight (L) measurements in male WT and male *Phb2^D/D^* mice. (M–R) Echocardiography was used to analyse the heart function. LV ESD, left ventricular end systolic diameter; LV EDD, left ventricular end diastolic diameter. (S) Histopathological analysis (H&E staining) of heart tissues from male WT and male *Phb2^D/D^* mice. (T) Analysis of myocardial fibrosis (Sirius red staining) in male WT and male *Phb2^D/D^* mice. (U) Western blot analysis of α‐SMA levels in heart tissues from male WT and male *Phb2^D/D^* mice. Experiments were repeated at least three times and the data are shown as mean ± SEM (N = three independent cell isolations per group). *p < .05.

To investigate the impact of Phb2 phosphorylation status on cardiac pathology in ACM, we created a transgenic mouse model expressing the *Phb2^S91D^* variant, mimicking constitutive dephosphorylation at serine 91. Homozygous male *Phb2^D/D^* mice grew and developed normally and showed high expression of p‐Phb2^S91^ in the heart (Figure 7A and 7B). The knockin mutation has no influence on liver/kidney/brain structure and function (Supplemental Figure S6A–E). Besides, no statistical significance was detected regarding the survival rate between male WT mice and *Phb2^D/D^* mice (Supplemental Figure S6F).

Following 8 weeks of ethanol exposure, no significant differences in caloric intake or body weight were observed between male WT and male *Phb2^D/D^* mice (Figure 7C–7E). However, ethanol‐treated WT males showed elevated levels of BNP and CM‐MB compared to *Phb2^D/D^* males (Figure 7F and 7G). Similarly, both serum and heart TG levels were markedly upregulated in male WT mice but not in male *Phb2^D/D^* mice (Figure 7H and 7I). EtOH treatment increased the heart weight, heart/body weight ratio and LV mass/body weight ratio, but these metrics were significantly restored to near‐normal levels in male *Phb2^D/D^* mice (Figure 7J–L). Additionally, echocardiography showed that EtOH impaired heart function in male WT mice, an impairment that was normalised in male *Phb2^D/D^* mice (Figure 7M–7R). Histological examination of heart tissues revealed that ethanol‐induced myocardial structural disarray (Figure 7S) was significantly attenuated in male *Phb2^D/D^* mice. Besides, EtOH‐mediated myocardial fibrosis was also attenuated in male *Phb2^D/D^* mice (Figure 7S–7U). These data thus confirmed that Phb2 dephosphorylation at Ser91 aggravates alcohol‐induced heart injury in mice.

To observe whether Phb2 phosphorylation improved heart performance in female mice, cardiac structure and function was measured upon EtOH treatment. Phb2 phosphorylation in heart tissues was markedly reduced by EtOH in female WT mice, whereas this alteration was attenuated in female *Phb2^D/D^* mice (Supplemental Figure S7A and S7B). However, augmentation of Phb2 phosphorylation slightly reduced EtOH‐caused BNP/CK‐MB upregulation (Supplemental Figure S7C and S7D). Besides, ADH/ALDH concentration and CYP2E1 activity were not significantly improved in female *Phb2^D/D^* mice upon EtOH treatment when compared with that in female WT mice (Supplemental Figure S7E and S7G). Further, histological analysis using HE staining (Supplemental Figure S7H) and Sirius red staining (Supplemental Figure S7I and S7J) also demonstrated that EtOH‐mediated cardiac injury was not markedly alleviated in female *Phb2^D/D^* mice, reconfirming that other signalling transduction pathways in addition to Pgam5/Phb2, is induced by alcohol in female mice.

### 3.9 *Phb2^S91D^* expression restores mitochondrial homeostasis and improves cell function in EtOH‐treated male mouse cardiomyocytes

Finally, we conducted experiments in neonatal cardiomyocytes isolated from male WT and male *Phb2^D/D^* mice to confirm whether inhibition of Phb2 dephosphorylation at Ser91 protects mitochondria and cardiomyocyte function against alcoholic injury in vitro. We found that after EtOH exposure, cellular viability was largely preserved in cardiomyocytes obtained from male *Phb2^D/D^* mice (Supplemental Figure S8A). Besides, the upregulation of pro‐inflammatory gene transcription (Supplemental Figure S8B–S8D), as well as the changes in ADH, ALDH and CYP2E1 expression (Supplemental Figure S8E–S8G) observed in EtOH‐exposed cardiomyocytes from male WT mice were largely prevented in male *Phb2^D/D^* cardiomyocytes.

Further suggesting a key role of Phb2^Ser91^ phosphorylation status in cardiomyocytes dysfunction associated with alcohol exposure, EtOH exposure failed to repress antioxidative enzyme expression (Supplemental Figure S8H and S8I), stimulate ROS production (Supplemental Figure S8J and S8K) and dissipate mitochondrial potential (Supplemental Figure S8L and S8M) in male *Phb2^D/D^* cardiomyocytes. Moreover, compared to male WT cells, EtOH‐related prolongation of the mPTP opening time was blunted in male *Phb2^D/D^* cardiomyocytes (Supplemental Figure S8N). These data thus confirmed that dephosphorylation of Phb2 at Ser91 is a key contributor to mitochondrial dysfunction and cardiomyocyte damage resulting from EtOH stress.

## 4 DISCUSSION

**FIGURE 8:**
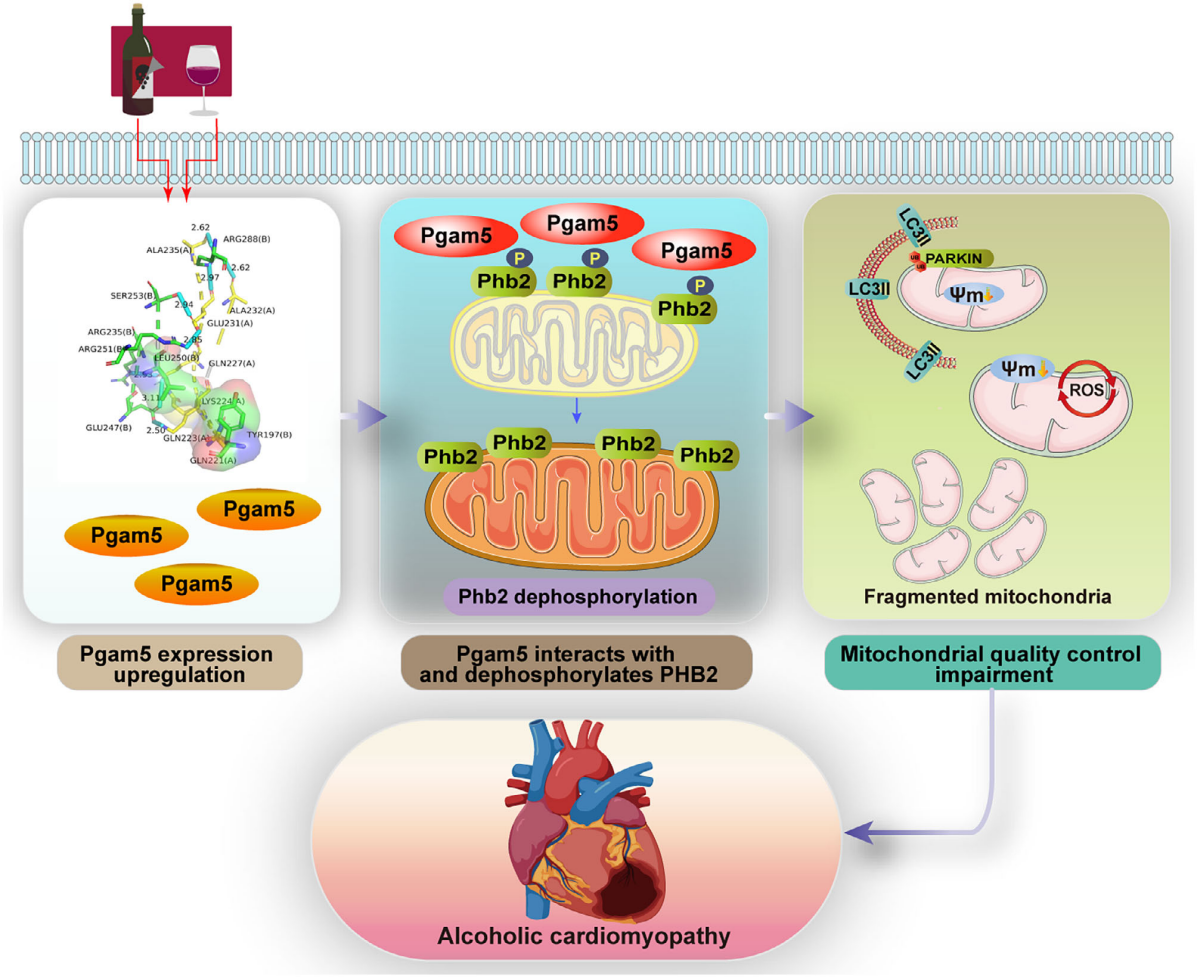
A schematic mechanism of Pgam5‐mediated PHB2 dephosphorylation in the pathogenesis of ACM.

Excessive alcohol consumption poses a significant threat to global health, ranking among the top contributors to premature death and disability globally. Uncovering new molecular targets could revolutionize our understanding of ACM's complex pathophysiology, paving the way for innovative therapies to halt or reverse the debilitating decline in cardiac function. Using gene knockout and knockin mice, our results identified Pgam5 as a pathological factor promoting alcohol‐caused myocardial injury in male mice. Specifically, we found that alcohol exposure promoted myocardial expression of Pgam5, whereas cardiomyocyte‐specific *Pgam5* KO prevented structural anomalies and sustained heart function through reducing inflammation and oxidative stress and improving cardiomyocyte metabolism in male mice. Molecular assays showed that alcohol exposure disrupted mitochondrial function, leading to impaired respiratory capacity, enhanced oxidative stress, dissipated membrane potential and increased mPTP opening. Notably, these alcohol‐mediated pathological changes in male mice heart could be reversed by *Pgam5* deletion through a mechanism involving normalisation of the MQC, characterised by reduced fission, recovered mitophagy and biogenesis and activation of mtUPR. Furthermore, using Co‐IP, western blotting, computational docking simulations and protein expression analyses, we revealed that alcohol exposure impaired MQC through inducing Pgam5‐mediated Phb2 dephosphorylation at Ser91. Accordingly, both disruption of Pgam5/Phb2 interaction and transduction of a Ser91 phospho‐mimetic mutant of Phb2 (Phb2^S91D^) were capable to maintain MQC, reduce mitochondrial disorder and improve myocyte function upon alcohol challenge. Moreover, knockin male mice carrying the *Phb2^S91D^* mutant gene showed resistance to alcohol‐related myocardial injury, characterised by the absence of fibrotic scar formation, dampened inflammation, reduced oxidative stress and enhanced cardiomyocyte function. These findings collectively reveal a previously unrecognised mechanism, wherein the Pgam5‐Phb2 complex plays a pivotal role in the development of ACM in males, shedding new light on the underlying pathophysiology (Figure 8). Hence, targeted therapies aimed at inhibiting Pgam5 and/or its binding to Phb2 may help preserve MQC in cardiomyocyte and thus alleviate clinical manifestations of ACM in male mice.

A wealth of research has conclusively implicated mitochondrial dysfunction as a key contributor to the development of alcohol‐induced cardiac damage, solidifying its status as a critical factor in the pathogenesis of ACM.^56^, ^57^, ^58^, ^59^ Multiple deleterious mitochondrial signals are activated in EtOH‐exposed cardiomyocytes, contributing to oxidative stress, abnormal lipid metabolism, inflammation and cell death.^60^, ^61^, ^62^, ^63^, ^64^ The MQC system is activated to relieve mitochondrial damage, promote selective degradation of damaged mitochondria and ultimately restore mitochondrial integrity and homeostasis. While previous studies have investigated the roles of fission,^65^ mitophagy^66^ and mitochondrial bioenergetics^67^ in ACM, other aspects governed by the MQC, such as the mtUPR and mitochondrial biogenesis, have not been thoroughly explored. Besides, it is unclear whether the various MQC processes are controlled by a common upstream signal. Here, we identified Phb2 dephosphorylation as an initial event mediating MQC dysfunction in response to EtOH treatment. Phb2 is an IMM‐associated scaffolding protein that forms a ring structure that protects mitochondrial membrane integrity. Increased Phb2 phosphorylation at Ser91 has been found to inhibit mitochondria‐mediated apoptosis and therefore to favour human leukaemia progression.^54^ Moreover, phosphorylation of Phb2 at Ser39 correlates with increased mitophagic activity due to enhanced interaction with LC3^55^ through an LC3‐interacting region (LIR) within Phb2.^68^ Besides, Phb2 autophosphorylation is thought to be a prerequisite for its translocation from the cytoplasm to the IMM,^69^ where it exerts well‐characterised anti‐apoptotic actions through preventing IMM hyper‐permeability.^70^ By comparison, decreased Phb2 phosphorylation is closely associated with activation of caspase‐mediated apoptosis.^71^ In line with this evidence, the present data from our mouse model of ACM confirmed that dephosphorylation of Phb2 at Ser91 is crucially linked to mitochondrial dysfunction and cardiomyocyte damage due to impaired MQC. These findings suggest that preservation of Phb2 phosphorylation status might be a relevant protective strategy to sustain cardiac performance during ACM.

Our previous studies indicated that Pgam5 plays a detrimental role in cardiac post‐ischemic damage through disrupting MQC.^72^ Supporting our present findings, the influence of Pgam5 on various cardiovascular conditions and diseases, such as heart failure,^17^ chronic doxorubicin‐mediated cardiotoxicity^73^ and LPS‐mediated myocardial injury,^74^ has been determined. Importantly, the pathological mechanisms underlying Pgam5‐mediated myocardial dysfunction are mainly associated with necroptosis, a form of inflammation‐related programmed cell death.^15^ However, recent studies have uncovered several molecular mechanisms by which Pgam5 modulates cell metabolism and mitochondrial integrity besides its role in necroptosis. Global *Pgam5* KO mice demonstrated enhanced cold tolerance and resistance to fasting‐induced stress, as evident from their ability to maintain body temperature and survive longer under these challenging conditions. These effects were due to decreased lipid accumulation in brown adipose tissue, resulting from elevated activity of UCP1 and accelerated OCR.^75^ A high‐fat diet led to heightened ROS production in liver mitochondria, which was accompanied by the dissociation of NFE2L2, a key transcriptional regulator of mitochondrial antioxidant defences, from its mitochondrial anchor Pgam5, impairing the cellular antioxidant response.^76^ In turn, another research reported Pgam5 upregulation promotes mitochondrial fragmentation and simultaneously inhibits mitophagy, leading to cellular senescence.^77^ These findings highlight the profound and complex influence of Pgam5 on cellular function besides its role in necroptosis regulation.

Our experiments describe a previously unknown mechanism of Pgam5 in ACM development, namely disruption of Phb2‐mediated MQC. We first determined that *Pgam5* deletion was able to reverse alcohol‐mediated myocardial alterations by normalising organ function and metabolism, preserving tissue structure and reducing inflammation and oxidative injury. Secondly, our in vitro data demonstrated that *Pgam5* ablation protected mitochondrial function and restored MQC‐associated mechanisms in the presence of EtOH. Lastly, through Co‐IP, western blotting and protein domain expression analyses, we showed that Pgam5 interacts with and dephosphorylates Phb2 at Ser91 in ethanol‐stressed neonatal cardiomyocytes, revealing a previously unknown mechanism by which Pgam5 contributes to mitochondrial dysfunction in ACM, thereby shedding new light on the underlying pathophysiology.

One limitation of our research is the lack of investigation into potential sex‐specific responses to alcohol, which may yield valuable insights into the complexities of ACM. Notably, our findings revealed that female WT mice exhibited more pronounced alcohol‐induced cardiac damage compared to their male counterparts. Moreover, unlike males, female mice lacking *Pgam5* in cardiomyocytes failed to show significant protection against ACM, hinting that other signalling transduction pathways, in addition to Pgam5/PHB2, are induced by alcohol in female mice and contributed to augmented live injury compared to male mice. These findings highlight the unresolved question regarding the divergent phenotypic alterations in male and female mice during alcohol exposure,^78^, ^79^, ^80^ calling for further research to address this question.

In summary, by modelling ACM in vivo in WT and in genetically modified mice, and through in vitro studies in their respective neonatal cardiomyocytes, we unmasked a pivotal contribution of Pgam5‐mediated Phb2 dephosphorylation to the development of alcohol‐related myocardial injury. Specifically, Phb2 dephosphorylation was found to destabilise MQC and impair cardiomyocyte function, metabolism and survival. These discoveries, coupled with the potential of Pgam5, Phb2 and MQC as tractable therapeutic targets, may lead to the development of novel, personalised treatment strategies for ACM, offering new hope for patients afflicted by this devastating disease.

## AUTHOR CONTRIBUTIONS

Jun Tao and Junxiong Qiu contributed to research designing. Junmeng Zheng, Ruibing Li, and Xing Chang conducted experiments. Ruibing Li and Xing Chang acquired and analysed data. Qingyong He and Jun Tao wrote the manuscript. Ruibing Li and Xing Chang contributed to the manuscript revision. All the authors approved the final manuscript.

## CONFLICT OF INTEREST STATEMENT

The authors declare no competing interests.

## ETHICAL APPROVAL

All procedures were in accordance with the ethical standards of the responsible committee on human experimentation (institutional and national) and the Helsinki Declaration of 1964 and later versions. This study was approved by the ethics committee of the Sun Yat‐sen Memorial Hospital (2022‐03‐T01).

## Supporting information

Supporting Information

## Data Availability

All data underlying the study's findings are contained within the manuscript, supplementary materials, and can be obtained from the corresponding author upon reasonable request.
