# Supplementary material for: Phosphoglycerate mutase 5 exacerbates alcoholic cardiomyopathy in male mice by inducing prohibitin‐2 dephosphorylation and impairing mitochondrial quality control
Source: Clin Transl Med. 2024 Aug 14;14(8):e1806. doi: 10.1002/ctm2.1806 (PMC11324691; doi:10.1002/ctm2.1806)
Supplement: Supplementary file 1 — Supporting Information [file CTM2-14-e1806-s001.docx]

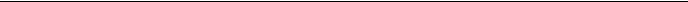

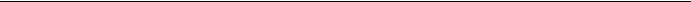
Supplemental tables and figures for

**Phosphoglycerate mutase 5 exacerbates alcoholic cardiomyopathy in male mice by inducing prohibitin-2 dephosphorylation and impairing mitochondrial quality control**

**Supplemental Table 1: Antibody information in Western blot**

| Name | Catalogue number | Dilution factor |
| --- | --- | --- |
| Drp1 | Abcam, #ab184247 | 1:1000 |
| Fis1 | Abcam, #ab156865 | 1:1000 |
| Mfn2 | Abcam, #ab124773 | 1:1000 |
| Opa1 | Abcam, #ab42364 | 1:1000 |
| GAPDH | Abcam, #ab8245 | 1:1000 |
| Tom20 | Abcam, #ab186735 | 1:1000 |
| PHB2 | Cell Signaling Technology, #14085 | 1:1000 |
| TnT | Abcam, #ab8295 | 1:1000 |
| Bcl-2 | Abcam, #ab182858 | 1:1000 |
| PKM2 | Abcam, #ab137791 | 1:1000 |
| Bax | Abcam, #ab3191 | 1:1000 |
| LC3II | Abcam, #ab192890 | 1:1000 |
| PGC1α | Abcam, #ab191838 | 1:1000 |
| Phb2 | Abcam, #ab75766 | 1:1000 |
| Bax | Abcam, #ab3191 | 1:1000 |
| IL-6 | Abcam, #ab290735 | 1:1000 |
| MMP9 | Abcam, #ab58803 | 1:1000 |
| Pgam5 | Abcam, #ab244218 | 1:1000 |
| Parkin | Abcam, #ab77924 | 1:1000 |
| p62 | Abcam, #ab91526 | 1:1000 |
| “-SMA | Cell Signaling Technology, #14968 | 1:1000 |

**Supplemental Table 2: Primers for qPCR**

Gene Forward Prime

Reverse Prime

*Pgc1α*

*Nrf2*

5′-CGGAAATCATATCCAACCAG-3′

5′-CCTCGCTGGAAAAAGAAGTG-3′

5′-TGAGGACCGCTAGCAAGTTTG-3′

5′-GGAGAGGATGCTGCTGAAAG-3′

| 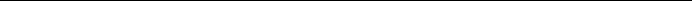*Tfam* | 5′-GGCGAATTCCTCGAGGCCACCATG  GCGCTGTTCCGGGGAATGT-3′ | 5′- CATACGCGTATGCTCAGAGATGTC  TCCGGATCGT -3′ |
| --- | --- | --- |
| *Gapdh* | 5′-ACGGCAAATTCAACGGCACAGTCA-3′ | 5′-TGGGGGCATCGGCAGAAGG-3′ |
| *mtHsp70* | 5′-CTCTGGGAGGCGTCTTTACC-3′ | 5′-CGTTCCCCCTGACACACTTT-3′ |
| *Phb2* | 5′-AGCAGGAACAGCACAGAAGA-3′ | 5′-CGGAGCTTGATATAGCCAGGAT-3′ |
| *Atf6* | 5′-CACAGCTCCCTAATCACGTGG-3′ | 5′-ACTGGGCTA TTCGCTGAAGG-3′ |
| *LonP1* | 5′-GGTTGAGAATGTAGCCCATGA-3′ | 5′-CGATGATATCCCGAATGGTC-3′ |
| *Pkm2* | 5′-GTCTGGAGAAACAGCCAAGG-3′ | 5′-CGGAGTTCCTCGAATAGCTG-3′ |
| *Mcp1* | 5'-GGATGGATTGCACAGCCATT-3 | 5'-GCGCCGACTCAGAGGTGT-3' |
| *Tnfα* | 5'-AGATGGAGCAACCTAAGGTC-3' | 5'-GCAGACCTCGCTGTTCTAGC-3' |
| *ClpP* | 5′-CACAGACATCGCCATCCA-3′ | 5′-TCCCTCTCCATTGCTGACTC-3′ |
| *Tgfβ* | 5′-ATACGTCAGACATTCGGGAAGCAGTG-3′ | 5′- AATAGTTGGTATCCAGGGCTCTCCG-3′ |
| *IL-6* | 5′-ACTCACCTCTTCAGAACGAATTG-3′ | 5′-CCATCTTTGGAAGGTTCAGGTTG-3′ |

**Supplemental Figures:**


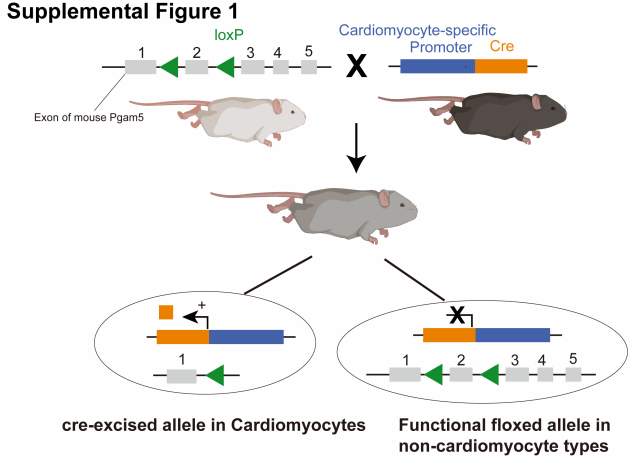


**Supplemental Figure 1.** A schematic of the construction of *Pgam5cKO* mice.


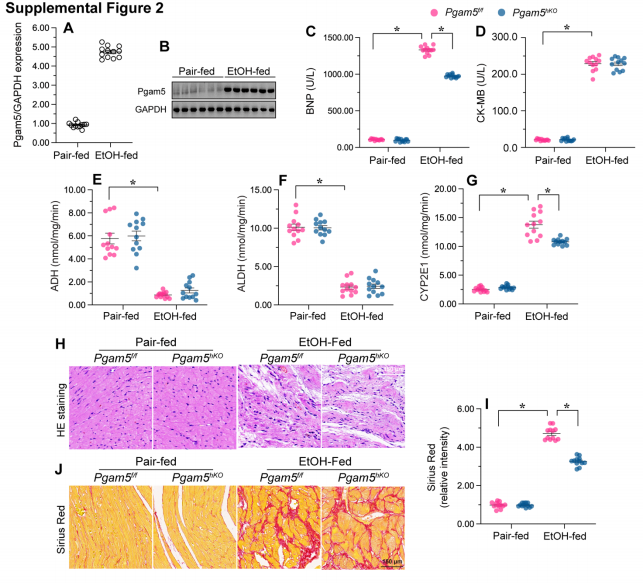


**Supplemental Figure 2.*Pgam5* KO fails to significantly improve heart function in a female mouse model of ACM.** Female wild-type (WT), female *Pgam5* knockout (*Pgam5cKO*), and female *Pgam5f/f* mice were pair- fed a liquid control or a 5% ethanol-containing diet for 8 weeks. **(A)** Western blot analysis of myocardial Pgam5 protein expression in female WT mice. **(C-G)** serum BNP (C), serum CK-MB (D), ADH (E), ALDH (F), and CYP2E1 (G) measurements in female *Pgam5cKO* and *Pgam5f/f* mice. **(H)** Histopathological analysis (H&E staining) of heart tissue from female *Pgam5cKO* and *Pgam5f/f* mice. **(I, J)** Analysis of myocardial fibrosis (picro-sirius red staining) in female *Pgam5cKO* and *Pgam5f/f* mice. *p<0.05.


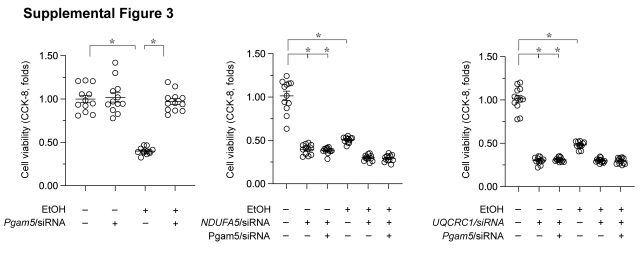


**Supplemental Figure 3. *Pgam5* KO reduces EtOH-caused cardiomyocyte death through preserving mitochondrial integrity.** Primary cardiomyocytes were isolated form WT mice and then transfected with *Pgam5*/siRNA, *NDUFA5*/siRNA, or *UQCRC1*/siRNA before EtOH treatment. Cell viability was measured via

CCK-8 assay. *p<0.05.


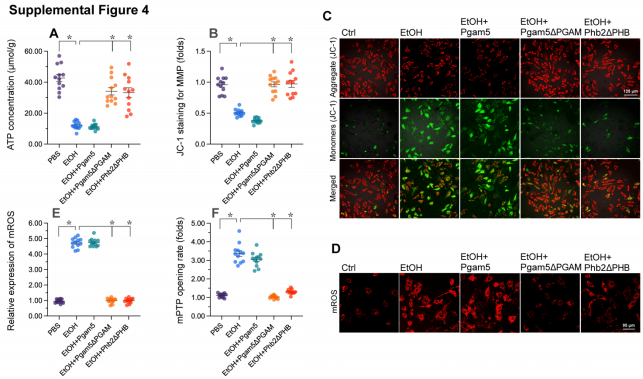


**Supplemental Figure 4. Transduction of Pgam5ΔPGAM or Phb2ΔPHB reduces EtOH-induced mitochondrial damage in HL-1 cells.** HL- 1 cells were transfected with a Pgam5 mutant lacking the PGAM domain (Pgam5ΔPGAM) or with a Phb2 mutant lacking the PHB domain (Phb2ΔPHB) prior to EtOH treatment. Full-length WT-Pgam5 was transduced as the control group. **(A)** ELISA-based analysis of mitochondrial ATP production in HL- 1 cells. **(B, C)** Analysis of mitochondrial membrane potential in HL- 1 cells loaded with JC- 1. **(D, E)** Representative images of HL- 1 cells loaded with the ROS indicator MitoSOX

Red. **(F)** TMRE-based analysis of mPTP opening rate in HL- 1 cells. *p<0.05.


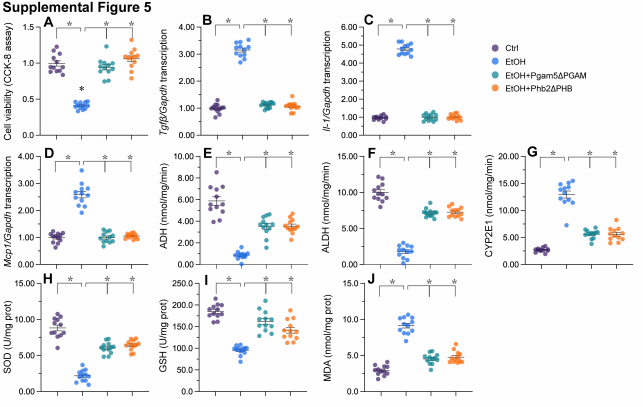


**Supplemental Figure 5. Pgam5ΔPGAM or Phb2ΔPHB expression attenuates EtOH-mediated HL-1 cells. (A).** Cell viability analysis (CCK-8 assay) in control and EtOH-treated HL- 1 cells transduced with Pgam5ΔPGAM or Phb2ΔPHB. **(B-D)** Transcriptional analysis of *Tgfβ, Il-1, and Mcp1* expression in HL- 1 cells transduced with Pgam5ΔPGAM or Phb2ΔPHB. **(E-G)** ELISA-based analysis of ADH, ALDH, and CYP2E1 levels in HL- 1 cells transduced with Pgam5ΔPGAM or Phb2ΔPHB. **(H-J)** ELISA-based analysis of

SOD, GSH, and MDA levels in HL- 1 cells transduced with Pgam5ΔPGAM or Phb2ΔPHB. *p<0.05.


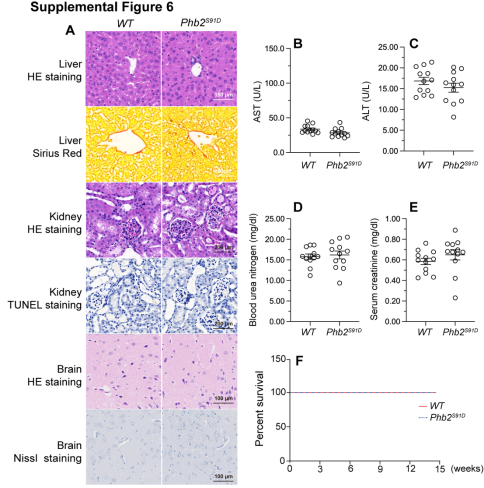


**Supplemental Figure 6. A knockin mutation in *Phb2S91D* has no influence on liver/kidney/brain structure and function. (A)** Liver, kidney and brain were isolated from male WT mice or male *Phb2D/D* mice. Then, HE staining, Sirius red staining, TUNEL staining, and Nissl staining were performed to observe the changes in liver, kidney and brain. **(B-C)** ELISA analysis of the concentration of ALT and AST in serum isolated from male WT mice or male *Phb2D/D* mice. **(D-E)** ELISA analysis of the concentration of blood urea nitrogen (BUN) and serum creatinine (Scr) in serum isolated from male WT mice or male *Phb2D/D* mice. **(F)** The survival rate of male male WT mice and male *Phb2D/D* mice. *p<0.05.


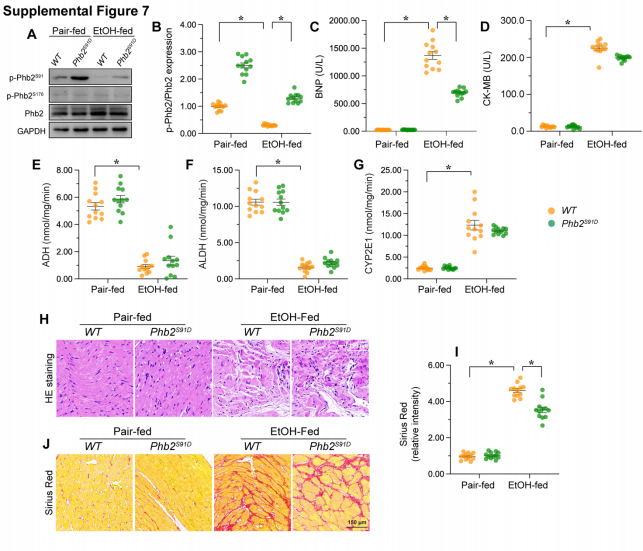


**Supplemental Figure 7. Reintroduction of Phb2 phosphorylation fails to significantly improve heart function in a female mouse model of ACM.** Female wild-type (WT) and female *Phb2D/D* mice were pair-fed a liquid control or a 5% ethanol-containing diet for 8 weeks. **(A-B)** Western blot analysis of p-Phb2S91 and total PHB2 levels in heart tissues from female WT or female *Phb2D/D* mice. **(C-G)** serum BNP (C), serum CK- MB (D), ADH (E), ALDH (F), and CYP2E1 (G) measurements in female WT or *Phb2D/D* mice. **(H)** Histopathological analysis (H&E staining) of heart tissue from female WT or female *Phb2D/D* mice. **(I, J)** Analysis of cardiac fibrosis (picro-sirius red staining) in female WT or female *Phb2D/D* mice. *p<0.05.


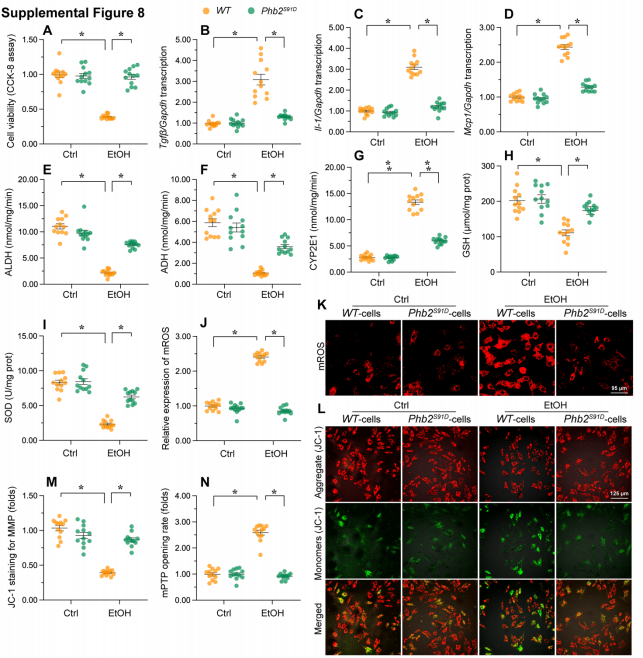


**Supplemental Figure 8. Expression of *Phb2S91D* sustains mitochondrial integrity and cardiomyocyte function after ETOH exposure.** Primary cardiomyocytes were isolated from male WT and homozygous male *Phb2S91D* (*Phb2D/D*) mice and treated with 100 mM ethanol for 48 h. **(A)** Cell viability analysis (CCK-8 assay) in neonatal cardiomyocytes. **(B-D)** Transcriptional analysis of *Tgfβ, Il-1, and Mcp1* expression in male mouse cardiomyocytes. **(E-G)** ELISA-based analysis of ADH, ALDH, and CYP2E1 levels in male mouse cardiomyocytes. **(H, I)** ELISA-based analysis of SOD and GSH levels in male mouse cardiomyocytes. **(J, K)** Representative images of mitochondrial ROS production in male mouse cardiomyocytes loaded with MitoSOX Red. **(L, M)** Analysis of mitochondrial membrane potential in male mouse cardiomyocytes loaded with JC- 1.

**(N)** TMRE-based analysis of mPTP opening rate in cultured male mouse cardiomyocytes. *p<0.05.
